# Supplementary material for: Goat Milk Nutritional Quality Software-Automatized Individual Curve Model Fitting, Shape Parameters Calculation and Bayesian Flexibility Criteria Comparison
Source: Animals (Basel). 2020 Sep 18;10(9):1693. doi: 10.3390/ani10091693 (PMC7552780; doi:10.3390/ani10091693)
Supplement: Supplementary file 1 [file animals-10-01693-s001.zip › Table S10.docx]

**Table S10:** Summary of curve shape parameters (b0, b1, b2, b3, b4 and knot), number of elements and flexibility selection criterion (RSS, AIC, AICc and BIC) for linear and non-linear models for milk lactose content in Murciano-Granadina goats.

| **Model name** | **b0** | **b1** | **b2** | **b3** | **b4** | **Knot** | **Elements** | **RSS** | **MSPE** | **AIC** | **AICc** | **BIC** |
| --- | --- | --- | --- | --- | --- | --- | --- | --- | --- | --- | --- | --- |
| Ali and Schaeffer model (ALISCH) | 4.79 | 0.00 | 0.00 | -0.37 | -0.10 | NA | 5 | 267.25 | 53.45 | 27.50 | 32.07 | 27.44 |
| Asymptotic Regression, Single Exponential decay to an arbitrary value (SXPDCY) | 5.02 | 0.00 | NA | NA | NA | NA | 2 | 270.97 | 135.49 | 27.59 | 32.16 | 27.54 |
| Asymptotic Regression, Lactation modification of Metcherlich Law of Diminishing Returns or Exponential growth model (METLAW) | 0.61 | -7.33 | 0.00 | -0.01 | NA | NA | 4 | 268.54 | 67.13 | 27.53 | 32.10 | 27.48 |
| Brody (BRODY) | 5.02 | 0.00 | 3.52 | NA | NA | NA | 3 | 270.94 | 90.31 | 27.59 | 32.16 | 27.54 |
| Cappio Borlino, biexponential (CAPBOR) | 5.11 | -0.01 | 0.00 | NA | NA | NA | 3 | 270.58 | 90.19 | 27.58 | 32.15 | 27.53 |
| Cobby and Le Du (COBLDU) | 6.01 | 0.00 | 0.00 | NA | NA | NA | 3 | 271.19 | 90.40 | 27.60 | 32.17 | 27.54 |
| Compound/ Exponential Growth (CEXPGR) | 14.38 | 1.00 | NA | NA | NA | NA | 2 | 6457.30 | 3228.65 | 49.79 | 54.36 | 49.74 |
| Cubic (CUBIC) | 5.05 | 0.00 | 0.00 | 0.00 | NA | NA | 4 | 268.10 | 67.02 | 27.52 | 32.09 | 27.46 |
| Cubic Spline function with one knot (CUBSPL) | -641.35 | 12.39 | -0.08 | 0.00 | 0.00 | 156.49 | 5 | 268.10 | 53.62 | 27.52 | 32.09 | 27.46 |
| Curve S (CURVES) | 1.57 | 0.19 | NA | NA | NA | NA | 2 | 303.86 | 151.93 | 28.39 | 32.97 | 28.34 |
| Density (DENSITY) | NC | NC | NC | NA | NA | NA | 3 | NC | NC | NC | NC | NC |
| Dhanoa (DHANOA) | 5.11 | 0.00 | 0.00 | NA | NA | NA | 3 | 269.30 | 89.77 | 27.55 | 32.12 | 27.50 |
| Dijkstra (DJKSTR) | -0.12 | 0.08 | 0.01 | -0.13 | NA | NA | 4 | 5181.20 | 1295.30 | 48.25 | 52.82 | 48.19 |
| Exponential decline function or Gaines (EDFGAIN) | 5.02 | 0.00 | NA | NA | NA | NA | 2 | 270.97 | 135.49 | 27.59 | 32.16 | 27.54 |
| Gauss (GAUSS) | 55.54 | 0.00 | 0.91 | NA | NA | NA | 3 | 278.39 | 92.80 | 27.78 | 32.35 | 27.73 |
| Gompertz (GMPRTZ) | 4.56 | -0.11 | 0.01 | NA | NA | NA | 3 | 268.88 | 89.63 | 27.54 | 32.11 | 27.48 |
| Grossman (GROSMN) | 5.12 | -0.01 | 0.00 | 0.00 | 0.00 | NA | 5 | 270.22 | 54.04 | 27.57 | 32.14 | 27.52 |
| Hayashi (HAYSHI) | -3590591119.23 | 13.03 | -0.19 | NA | NA | NA | 3 | 303.86 | 101.29 | 28.39 | 32.97 | 28.34 |
| Inverse quadratic polynomial (INVQPOL) | -9202.87 | 300.98 | -1.66 | NA | NA | NA | 3 | 71374.79 | 23791.60 | 66.61 | 71.18 | 66.55 |
| Inverse, linear Hyperbolic (INVLINHY) | 4.79 | 1.02 | NA | NA | NA | NA | 2 | 303.38 | 151.69 | 28.38 | 32.95 | 28.33 |
| Johnson Schumacher (JOHNSCH) | 4.81 | 0.01 | -75.33 | NA | NA | NA | 3 | 309.83 | 103.28 | 28.53 | 33.10 | 28.48 |
| Log Logistic (LOGLOG) | 3.97 | -1.06 | -0.08 | NA | NA | NA | 3 | 0.00 | 0.00 | NA | NA | NA |
| Log Modified Weibull (LGMWEIB) | -5824.29 | 114.67 | 1.90 | NA | NA | NA | 3 | 0.00 | 0.00 | NA | NA | NA |
| Logarithmic (LOGARITH) | 5.37 | -0.12 | NA | NA | NA | NA | 2 | 274.74 | 137.37 | 27.69 | 32.26 | 27.64 |
| Madalena (MADALN) | 5.01 | 0.00 | NA | NA | NA | NA | 2 | 271.19 | 135.60 | 27.60 | 32.17 | 27.54 |
| Michaelis Menten (MICHMEN) | NA | NC | NC | NA | NA | NA | 2 | NC | NC | NC | NC | NC |
| MilkBot (MILKBOT) | 113.04 | 0.02 | 0.00 | NA | NA | NA | 3 | 72094.77 | 24031.59 | 66.68 | 71.25 | 66.62 |
| Molina and Boschini/Modal Linear (MOL&BOS) | 4.65 | 0.00 | 199.48 | NA | NA | NA | 3 | 269.37 | 89.79 | 27.55 | 32.12 | 27.50 |
| Morgan Mercer Florin (MORMFLO) | 4.67 | 0.00 | 5.02 | -2.99 | NA | NA | 4 | 267.47 | 66.87 | 27.50 | 32.07 | 27.45 |
| Nelder, inverser polynomial, Yadav (NELDER) | -9956.35 | 319.22 | -1.75 | NA | NA | NA | 3 | 71312.76 | 23770.92 | 66.60 | 71.17 | 66.55 |
| Parabolic exponential model and Parabolic, Sikka (PEMSIK) | 5.08 | 0.00 | 0.00 | NA | NA | NA | 3 | 268.49 | 89.50 | 27.53 | 32.10 | 27.47 |
| Parabolic yield-density (PARYLDENS) | -1862.74 | 30.06 | -0.12 | NA | NA | NA | 3 | 71621.16 | 23873.72 | 66.63 | 71.20 | 66.58 |
| Power (POWER) | 5.39 | 0.03 | NA | NA | NA | NA | 2 | 275.23 | 137.61 | 27.70 | 32.27 | 27.65 |
| Quadratic cum log model (QDCMLOG) | 4.87 | -0.01 | 0.00 | 0.09 | NA | NA | 4 | 267.30 | 66.82 | 27.50 | 32.07 | 27.44 |
| Quadratic model (QUADRT) | 5.08 | 0.00 | 0.00 | NA | NA | NA | 3 | 268.45 | 89.48 | 27.53 | 32.10 | 27.47 |
| Quadratic model Dave (DAVE) | 5.08 | 0.00 | 0.00 | NA | NA | NA | 3 | 268.45 | 89.48 | 27.53 | 32.10 | 27.47 |
| Quadratic spline function with one knot (QUADSPL) | 5.07 | 0.00 | 0.00 | 0.00 | NA | 7.77 | 4 | 268.45 | 67.11 | 27.53 | 32.10 | 27.47 |
| Ratio Cubics/Partial Fraction with Cubic Denominator (RATCUB) | 3.35 | -4.64 | 1.35 | 1.04 | 0.22 | NA | 5 | 284.23 | 56.85 | 27.93 | 32.50 | 27.87 |
| Ratio Quadratics/Partial Fraction with Quadratic Denominator (RATQUAD) | 0.00 | 0.00 | 0.00 | 0.00 | NA | NA | 4 | 295.29 | 73.82 | 28.19 | 32.77 | 28.14 |
| Richards (RICHRDS) | 4.69 | 0.05 | 998.33 | NA88.87 | NA | NA | 4 | 268.16 | 67.04 | 27.52 | 32.09 | 27.47 |
| Rook (ROOK) | 0.00 | 3.91 | 0.00 | 0.00 | NA | NA | 4 | 270.97 | 67.74 | 27.59 | 32.16 | 27.54 |
| Simple Linear (SIMLIN) | 5.01 | 0.00 | NA | NA | NA | NA | 2 | 271.19 | 135.60 | 27.60 | 32.17 | 27.54 |
| Singh And Gopal (SIN&GOP) | 5.12 | 0.00 | -0.04 | NA | NA | NA | 3 | 270.67 | 90.22 | 27.59 | 32.16 | 27.53 |
| Third order Legendre ortogonal polynomial (3ORDLEG) | -166.09 | -334.91 | -153.05 | -144.37 | NA | NA | 4 | 16087.31 | 4021.83 | 56.18 | 60.75 | 56.13 |
| Verhulst/Logistic differential equation/Pearl Reed (VERHLST) | 4.55 | -0.10 | 0.01 | NA | NA | NA | 3 | 268.91 | 89.64 | 27.54 | 32.11 | 27.49 |
| Von Bertalanffy (VBRTLNFY) | 4.56 | -0.04 | 0.01 | NA | NA | NA | 3 | 268.88 | 89.63 | 27.54 | 32.11 | 27.48 |
| Weibull, Parametric Survival Models (PARSURW) | 185.69 | 99.28 | -3555.54 | 1269.06 | NA | NA | 4 | 0.00 | 0.00 | NA | NA | NA |
| Wilmink’s exponential (WILMINK) | 4.99 | 0.09 | 0.00 | NA | NA | NA | 3 | 270.86 | 90.29 | 27.59 | 32.16 | 27.54 |
| Wood (WOOD) | 4.99 | -0.09 | NA | 0.00 | NA | NA | 3 | 270.86 | 90.29 | 27.59 | 32.16 | 27.54 |
| NC: Does not converge, NA: Does not apply. | | | | | | | | | | | | |
