# Supplementary material for: Goat Milk Nutritional Quality Software-Automatized Individual Curve Model Fitting, Shape Parameters Calculation and Bayesian Flexibility Criteria Comparison
Source: Animals (Basel). 2020 Sep 18;10(9):1693. doi: 10.3390/ani10091693 (PMC7552780; doi:10.3390/ani10091693)
Supplement: Supplementary file 1 [file animals-10-01693-s001.zip › Table S11.docx]

**Table S11:** Summary of curve shape parameters (b0, b1, b2, b3, b4 and knot), number of elements and flexibility selection criterion (RSS, AIC, AICc and BIC) for linear and non-linear models for milk somatic cells count in Murciano-Granadina goats.

| **Model name** | **b0** | **b1** | **b2** | **b3** | **b4** | **Knot** | **Elements** | **RSS** | **MSPE** | **AIC** | **AICc** | **BIC** |
| --- | --- | --- | --- | --- | --- | --- | --- | --- | --- | --- | --- | --- |
| Ali and Schaeffer model (ALISCH) | 446.13 | 17.16 | -0.02 | -1419.63 | -1029.22 | NA | 5 | 4421915309.35 | 884383061.87 | 143.85 | 148.42 | 143.79 |
| Asymptotic Regression, Single Exponential decay to an arbitrary value (SXPDCY) | 701.59 | 0.00 | NA | NA | NA | NA | 2 | 4448085877.12 | 2224042938.56 | 143.89 | 148.46 | 143.83 |
| Asymptotic Regression, Lactation modification of Metcherlich Law of Diminishing Returns or Exponential growth model (METLAW) | 383.50 | -1.90 | 0.03 | -0.01 | NA | NA | 4 | 4423196493.63 | 1105799123.41 | 143.85 | 148.42 | 143.80 |
| Brody (BRODY) | 701.70 | 0.00 | 2.86 | NA | NA | NA | 3 | 4448074427.23 | 1482691475.74 | 143.89 | 148.46 | 143.83 |
| Cappio Borlino, biexponential (CAPBOR) | 1450.29 | -0.23 | 0.00 | NA | NA | NA | 3 | 4430041231.98 | 1476680410.66 | 143.86 | 148.43 | 143.81 |
| Cobby and Le Du (COBLDU) | 706.02 | -0.81 | 4.76 | NA | NA | NA | 3 | 4448979316.03 | 1482993105.34 | 143.89 | 148.46 | 143.84 |
| Compound/ Exponential Growth (CEXPGR) | 701.58 | 1.00 | - | NA | NA | NA | 2 | 4448085876.92 | 2224042938.46 | 143.89 | 148.46 | 143.83 |
| Cubic (CUBIC) | 1035.10 | -9.84 | 0.08 | 0.00 | NA | NA | 4 | 4423610897.21 | 1105902724.30 | 143.85 | 148.42 | 143.80 |
| Cubic Spline function with one knot (CUBSPL) | 676.40 | 0.45 | -0.02 | 0.00 | 0.00 | 104.64 | 5 | 4423610897.23 | 884722179.45 | 143.85 | 148.42 | 143.80 |
| Curve S (CURVES) | 6.69 | 0.33 | NA | NA | NA | NA | 2 | 4457834122.49 | 2228917061.24 | 143.90 | 148.48 | 143.85 |
| Density (DENSITY) | NC | NC | NC | NA | NA | NA | 3 | NC | NC | NC | NC | NC |
| Dhanoa (DHANOA) | 1060.40 | 0.01 | 0.03 | NA | NA | NA | 3 | 4427046423.70 | 1475682141.23 | 143.86 | 148.43 | 143.80 |
| Dijkstra (DJKSTR) | 1112.30 | -0.02 | 0.01 | -3.04 | NA | NA | 4 | 4423636449.65 | 1105909112.41 | 143.85 | 148.42 | 143.80 |
| Exponential decline function or Gaines (EDFGAIN) | 701.59 | 0.00 | NA | NA | NA | NA | 2 | 4448085877.16 | 2224042938.58 | 143.89 | 148.46 | 143.83 |
| Gauss (GAUSS) | 805.68 | -45.33 | 153.22 | NA | NA | NA | 3 | 4458443726.45 | 1486147908.82 | 143.91 | 148.48 | 143.85 |
| Gompertz (GMPRTZ) | 1448.78 | -0.23 | 0.00 | 0.02 | -0.01 | NA | 3 | 4429642486.79 | 1476547495.60 | 143.86 | 148.43 | 143.81 |
| Grossman (GROSMN) | 80143438.50 | 2176.21 | -0.33 | NA | NA | NA | 5 | 4457834206.57 | 891566841.31 | 143.90 | 148.48 | 143.85 |
| Hayashi (HAYSHI) | -1104.05 | 15.89 | -0.06 | NA | NA | NA | 3 | 6498911774.03 | 2166303924.68 | 146.54 | 151.11 | 146.49 |
| Inverse quadratic polynomial (INVQPOL) | 799.33 | 334.78 | NA | NA | NA | NA | 3 | 4457737967.41 | 1485912655.80 | 143.90 | 148.48 | 143.85 |
| Inverse, linear Hyperbolic (INVLINHY) | 746.21 | 9.70 | -290.34 | NA | NA | NA | 2 | 4412638312.40 | 2206319156.20 | 143.83 | 148.40 | 143.78 |
| Johnson Schumacher (JOHNSCH) | 805.68 | -129.66 | 34.50 | NA | NA | NA | 3 | 4458443726.45 | 1486147908.82 | 143.91 | 148.48 | 143.85 |
| Log Logistic (LOGLOG) | 4961.48 | 96.31 | 31.33 | NA | NA | NA | 3 | 0.00 | 0.00 | NA | NA | NA |
| Log Modified Weibull (LGMWEIB) | 727.57 | 17.14 | NA | NA | NA | NA | 3 | 4457765420.50 | 1485921806.83 | 143.90 | 148.48 | 143.85 |
| Logarithmic (LOGARITH) | 706.02 | -0.81 | NA | NA | NA | NA | 2 | 4448979320.80 | 2224489660.40 | 143.89 | 148.46 | 143.84 |
| Madalena (MADALN) | NC | NC | NA | NA | NA | NA | 2 | NC | NC | NC | NC | NC |
| Michaelis Menten (MICHMEN) | 14280.19 | 0.02 | 0.00 | NA | NA | NA | 2 | 6474229059.05 | 884383061.87 | 146.52 | 151.09 | 146.46 |
| MilkBot (MILKBOT) | 1877.90 | 0.81 | 1446.19 | NA | NA | NA | 3 | 4448979320.80 | 1482993106.93 | 143.89 | 148.46 | 143.84 |
| Molina and Boschini/Modal Linear (MOL&BOS) | 805.83 | 39.37 | -8991.60 | -3061.17 | NA | NA | 3 | 4458324895.25 | 1486108298.42 | 143.90 | 148.48 | 143.85 |
| Morgan Mercer Florin (MORMFLO) | -1104.19 | 15.90 | -0.06 | NA | NA | NA | 4 | 6498902899.85 | 1624725724.96 | 146.54 | 151.11 | 146.49 |
| Nelder, inverser polynomial, Yadav (NELDER) | 893.63 | 0.00 | 0.00 | NA | NA | NA | 3 | 4431611996.58 | 1477203998.86 | 143.86 | 148.43 | 143.81 |
| Parabolic exponential model and Parabolic, Sikka (PEMSIK) | -0.01 | 0.00 | 0.00 | NA | NA | NA | 3 | 6433692647.47 | 2144564215.82 | 146.47 | 151.04 | 146.42 |
| Parabolic yield-density (PARYLDENS) | 717.39 | 0.03 | 0.00 | NA | NA | NA | 3 | 4457648696.84 | 1485882898.95 | 143.90 | 148.48 | 143.85 |
| Power (POWER) | 1265.90 | 0.78 | 0.01 | -153.58 | NA | NA | 2 | 4427056993.83 | 2213528496.92 | 143.86 | 148.43 | 143.80 |
| Quadratic cum log model (QDCMLOG) | 894.45 | -3.62 | 0.02 | NA | NA | NA | 4 | 4430461258.30 | 1107615314.58 | 143.86 | 148.43 | 143.81 |
| Quadratic model (QUADRT) | 894.45 | -3.62 | -0.02 | NA | NA | NA | 3 | 4430461258.30 | 1476820419.43 | 143.86 | 148.43 | 143.81 |
| Quadratic model Dave (DAVE) | 592.41 | -5.10 | 0.02 | 0.00 | NA | -408.30 | 3 | 4430461258.36 | 1476820419.45 | 143.86 | 148.43 | 143.81 |
| Quadratic spline function with one knot (QUADSPL) | -0.81 | 0.60 | 0.12 | 0.27 | 0.00 | NA | 4 | 4456640890.29 | 1114160222.57 | 143.90 | 148.47 | 143.85 |
| Ratio Cubics/Partial Fraction with Cubic Denominator (RATCUB) | -19.99 | 17.03 | 15.33 | 0.02 | NA | NA | 5 | 4456910453.81 | 891382090.76 | 143.90 | 148.47 | 143.85 |
| Ratio Quadratics/Partial Fraction with Quadratic Denominator (RATQUAD) | 224.69 | -5.85 | -0.01 | -0.01 | NA | NA | 4 | 0.00 | 0.00 | NA | NA | NA |
| Richards (RICHRDS) | 0.02 | -0.26 | 0.00 | 0.00 | NA | NA | 4 | 4448086745.66 | 1112021686.41 | 143.89 | 148.46 | 143.83 |
| Rook (ROOK) | 706.02 | 0.81 | NA | NA | NA | NA | 4 | 4448979320.80 | 1112244830.20 | 143.89 | 148.46 | 143.84 |
| Simple Linear (SIMLIN) | 1405.95 | -3.35 | -222.23 | NA | NA | NA | 2 | 4428089511.16 | 2214044755.58 | 143.86 | 148.43 | 143.80 |
| Singh And Gopal (SIN&GOP) | 463.97 | -582.82 | -584.57 | -282.80 | NA | NA | 3 | 4448401778.12 | 1482800592.71 | 143.89 | 148.46 | 143.84 |
| Third order Legendre ortogonal polynomial (3ORDLEG) | 805.68 | -46.94 | 157.49 | NA | NA | NA | 4 | 4458443726.45 | 1114610931.61 | 143.91 | 148.48 | 143.85 |
| Verhulst/Logistic differential equation/Pearl Reed (VERHLST) | 805.68 | -3.28 | 22.68 | NA | NA | NA | 3 | 4458443726.45 | 1486147908.82 | 143.91 | 148.48 | 143.85 |
| Von Bertalanffy (VBRTLNFY) | 562.00 | -243.83 | 5133.00 | -1958610.36 | NA | NA | 3 | 4458324892.57 | 1486108297.52 | 143.90 | 148.48 | 143.85 |
| Weibull, Parametric Survival Models (PARSURW) | 528.97 | 709.16 | 1.85 | NA | NA | NA | 4 | 4426063085.62 | 1106515771.41 | 143.85 | 148.43 | 143.80 |
| Wilmink’s exponential (WILMINK) | 528.97 | -709.16 | NA | -1.85 | NA | NA | 3 | 4426063085.62 | 1475354361.87 | 143.85 | 148.43 | 143.80 |
| Wood (WOOD) | NC | NC | NC | NA | NA | NA | 3 | NC | NC | NC | NC | NC |
| NC: Does not converge, NA: Does not apply. | | | | | | | | | | | | |
