# Supplementary material for: Goat Milk Nutritional Quality Software-Automatized Individual Curve Model Fitting, Shape Parameters Calculation and Bayesian Flexibility Criteria Comparison
Source: Animals (Basel). 2020 Sep 18;10(9):1693. doi: 10.3390/ani10091693 (PMC7552780; doi:10.3390/ani10091693)
Supplement: Supplementary file 1 [file animals-10-01693-s001.zip › Table S12.docx]

**Table S12:** Summary of Bayesian ANOVA to test for differences in the mean for AIC across models comprising two, three, four or five elements.

|  | **Protein**  **(%)** | **Fat**  **(%)** | **Dry Matter**  **(%)** | **Lactose**  **(%)** | **Somatic cells count**  **(sc/mL)** |
| --- | --- | --- | --- | --- | --- |
| Sum of Squares | 223.113 | 81.901 | 435.081 | 276.613 | 0.270 |
| df | 3 | 3 | 3 | 3 | 3 |
| Mean Square | 74.371 | 27.300 | 145.027 | 92.204 | 0.090 |
| F | 0.970 | 0.581 | 1.587 | 0.581 | 0.144 |
| Sig. | 0.416 | 0.631 | 0.207 | 0.631 | 0.933 |
| Bayes Factor | 0.019 | 0.011 | 0.042 | 0.011 | 0.006 |
| 2 elements models Posterior Mean | 36.208 | 53.272 | 48.122 | 30.259 | 144.170 |
| 2 elements model 95CI | 30.310-42.105 | 48.662-57.883 | 41.691-54.553 | 21.774-38.744 | 143.637-144.703 |
| 3 elements models Posterior Mean | 40.875 | 55.145 | 55.708 | 35.099 | 144.134 |
| 3 elements model 95CI | 36.918-44.831 | 52.197-58.094 | 51.594-59.821 | 29.544-40.653 | 143.785-144.483 |
| 4 elements models Posterior Mean | 37.935 | 52.744 | 51.750 | 32.531 | 144.139 |
| 4 elements model 95CI | 32.600-43.269 | 48.573-56.914 | 45.933-57.567 | 24.482-40.58 | 143.633-144.645 |
| 5 elements models Posterior Mean | 34.623 | 51.180 | 49.633 | 27.630 | 143.875 |
| 5 elements model 95CI | 25.776-43.469 | 44.264-58.096 | 39.986-59.279 | 14.903-40.357 | 143.075-144.675 |
