# Supplementary material for: Goat Milk Nutritional Quality Software-Automatized Individual Curve Model Fitting, Shape Parameters Calculation and Bayesian Flexibility Criteria Comparison
Source: Animals (Basel). 2020 Sep 18;10(9):1693. doi: 10.3390/ani10091693 (PMC7552780; doi:10.3390/ani10091693)
Supplement: Supplementary file 1 [file animals-10-01693-s001.zip › Table S13.docx]

**Table S13:** Summary of Bayesian ANOVA to test for differences in the mean for AICc across models comprising two, three, four or five elements.

|  | **Protein**  **(%)** | **Fat**  **(%)** | **Dry Matter**  **(%)** | **Lactose**  **(%)** | **Somatic cells count**  **(sc/mL)** |
| --- | --- | --- | --- | --- | --- |
| Sum of Squares | 223.064 | 82.069 | 435.081 | 276.600 | 0.269 |
| df | 3 | 3 | 3 | 3 | 3 |
| Mean Square | 74.355 | 27.356 | 145.027 | 92.200 | 0.090 |
| F | 0.970 | 0.582 | 1.587 | 0.581 | 0.143 |
| Sig. | 0.416 | 0.630 | 0.207 | 0.631 | 0.934 |
| Bayes Factor | 0.019 | 0.011 | 0.042 | 0.011 | 0.006 |
| 2 elements models Posterior Mean | 40.779 | 48.644 | 48.122 | 34.830 | 148.741 |
| 2 elements model 95CI | 34.881-46.676 | 44.034-53.255 | 41.691-54.553 | 26.345-43.315 | 148.208-149.274 |
| 3 elements models Posterior Mean | 45.445 | 50.521 | 55.708 | 39.669 | 148.707 |
| 3 elements model 95CI | 41.488-49.401 | 47.573-53.47 | 51.594-59.821 | 34.115-45.223 | 148.358-149.056 |
| 4 elements models Posterior Mean | 42.505 | 48.117 | 51.750 | 37.102 | 148.710 |
| 4 elements model 95CI | 37.17-47.839 | 43.947-52.287 | 45.933-57.567 | 29.053-45.151 | 148.204-149.216 |
| 5 elements models Posterior Mean | 39.193 | 46.553 | 49.633 | 32.200 | 148.448 |
| 5 elements model 95CI | 30.346-48.039 | 39.637-53.468 | 39.986-59.279 | 19.473-44.927 | 147.648-149.247 |
