# Supplementary material for: Goat Milk Nutritional Quality Software-Automatized Individual Curve Model Fitting, Shape Parameters Calculation and Bayesian Flexibility Criteria Comparison
Source: Animals (Basel). 2020 Sep 18;10(9):1693. doi: 10.3390/ani10091693 (PMC7552780; doi:10.3390/ani10091693)
Supplement: Supplementary file 1 [file animals-10-01693-s001.zip › Table S14.docx]

**Table S14:** Summary of Bayesian ANOVA to test for differences in the mean for Adjusted BIC across models comprising two, three, four or five elements.

|  | **Protein**  **(%)** | **Fat**  **(%)** | **Dry Matter**  **(%)** | **Lactose**  **(%)** | **Somatic cells count**  **(sc/mL)** |
| --- | --- | --- | --- | --- | --- |
| Sum of Squares | 223.137 | 81.849 | 434.711 | 276.649 | 0.267 |
| df | 3.000 | 3.000 | 3.000 | 3.000 | 3.000 |
| Mean Square | 74.379 | 27.283 | 144.904 | 92.216 | 0.089 |
| F | 0.971 | 0.581 | 1.586 | 0.581 | 0.142 |
| Sig. | 0.416 | 0.631 | 0.207 | 0.631 | 0.934 |
| Bayes Factor | 0.019 | 0.011 | 0.042 | 0.011 | 0.006 |
| 2 elements models Posterior Mean | 36.154 | 48.702 | 48.072 | 30.207 | 144.113 |
| 2 elements model 95CI | 30.258-42.051 | 44.092-53.312 | 41.642-54.503 | 21.722-38.691 | 143.58-144.647 |
| 3 elements models Posterior Mean | 40.820 | 50.575 | 55.654 | 35.044 | 144.081 |
| 3 elements model 95CI | 36.865-44.776 | 47.626-53.523 | 51.541-59.767 | 29.49-40.599 | 143.732-144.431 |
| 4 elements models Posterior Mean | 37.879 | 48.174 | 51.695 | 32.477 | 144.087 |
| 4 elements model 95CI | 32.545-43.213 | 44.004-52.344 | 45.879-57.512 | 24.428-40.526 | 143.581-144.593 |
| 5 elements models Posterior Mean | 34.568 | 46.610 | 49.580 | 27.573 | 143.823 |
| 5 elements model 95CI | 25.722-43.413 | 39.695-53.525 | 39.934-59.226 | 14.846-40.299 | 143.022-144.623 |
