# Supplementary material for: Goat Milk Nutritional Quality Software-Automatized Individual Curve Model Fitting, Shape Parameters Calculation and Bayesian Flexibility Criteria Comparison
Source: Animals (Basel). 2020 Sep 18;10(9):1693. doi: 10.3390/ani10091693 (PMC7552780; doi:10.3390/ani10091693)
Supplement: Supplementary file 1 [file animals-10-01693-s001.zip › Table S15.docx]

**Table S15:** Summary of posterior distribution statistics and 95% credibility interval for milk protein, fat, dry matter, lactose (%) and somatic cells count (sc/mL) peaks and persistencies.

| Trait | Parameter | Posterior | | | 95% Credible Interval | |
| --- | --- | --- | --- | --- | --- | --- |
|  |  | Mode | Mean | Variance | Lower Bound | Upper Bound |
| Protein (%) | Peak | 38.480 | 50.320 | 460.377 | 23.798 | 104.462 |
|  | Persistence (b_1_) | 0.003 | 0.004 | 0.000 | 0.002 | 0.008 |
|  | Persistence (b_2_) | 0.000 | 0.000 | 0.000 | 0.000 | 0.000 |
|  | % | 0.174 | 0.174 | 0.000 | 0.166 | 0.183 |
| Fat (%) | Peak | 2231.833 | 2918.551 | 1548715.841 | 1380.261 | 6058.819 |
|  | Persistence (b_1_) | 0.051 | 0.066 | 0.001 | 0.031 | 0.138 |
|  | Persistence (b_2_) | 0.000 | 0.000 | 0.000 | 0.000 | 0.000 |
|  | % | 1.033 | 1.034 | 0.001 | 0.983 | 1.088 |
| Dry matter  (%) | Peak | 2289.127 | 2993.473 | 1629251.528 | 1415.694 | 6214.356 |
|  | Persistence (b_1_) | 0.062 | 0.081 | 0.001 | 0.038 | 0.168 |
|  | Persistence (b_2_) | 0.000 | 0.000 | 0.000 | 0.000 | 0.000 |
|  | % | 1.535 | 1.537 | 0.002 | 1.461 | 1.617 |
| Lactose (%) | Peak | 30.528 | 39.922 | 289.772 | 18.880 | 82.876 |
|  | Persistence (b_1_) | 0.002 | 0.002 | 0.000 | 0.001 | 0.005 |
|  | Persistence (b_2_) | 0.000 | 0.000 | 0.000 | 0.000 | 0.000 |
|  | % | 0.073 | 0.073 | 0.000 | 0.069 | 0.076 |
| Somatic cell counts (sc/mL) | Peak | 5521874.299 | 7220912.55 | 9480286906765 | 3414963.67 | 14990386.06 |
|  | Persistence (b_1_) | 167473036 | 2190032021 | 87204368312142 | 1035725021 | 4546437209 |
|  | sc/mL | 900945.47 | 902139.570 | 539692177.67 | 857743.596 | 948800.005 |
