# Supplementary material for: Goat Milk Nutritional Quality Software-Automatized Individual Curve Model Fitting, Shape Parameters Calculation and Bayesian Flexibility Criteria Comparison
Source: Animals (Basel). 2020 Sep 18;10(9):1693. doi: 10.3390/ani10091693 (PMC7552780; doi:10.3390/ani10091693)
Supplement: Supplementary file 1 [file animals-10-01693-s001.zip › Table S2.docx]

**Table S2:** SPSS models syntax for curve modellization in SPSS.

| **Model Name** | **SPSS model syntax** |
| --- | --- |
| Ali and Schaeffer model (ALISCH) | b0 + b1 * days + b2 * (days ** 2 ) + b3 * (lg10 (1/days)) + b4 * (lg10 (1/days) ** 2) |
| Asymptotic Regression, Single Exponential decay to an arbitrary value (SXPDCY) | b0 * (1 - b1) ** days |
| Asymptotic Regression, Lactation modification of Metcherlich Law of Diminishing Returns or Exponential growth model (METLAW) | b0 * (1 - b1 * exp (-b2 * days) - (b3 * days)) |
| Brody (BRODY) | b0*Exp(-b1*days) - b0*Exp(-b2*days) |
| Cappio Borlino, biexponential (CAPBOR) | b0 * days ** b1 * Exp(- b2 * days) |
| Cobby and Le Du (COBLDU) | b0*1-Exp(-b2 * days)-(b1*days) |
| Compound/ Exponential Growth (CEXPGR) | b0 * (b1**days) |
| Cubic (CUBIC) | b0 + (b1 * days) + (b2 * days**2) + (b3 * days**3) |
| Cubic Spline function with one knot (CUBSPL) | b0+ b1 * days + b2 * days ** 2 + b3 * (days ) ** 3+ b4 * (days - Knot) ** 3 |
| Curve S (CURVES) | Exp(b0 + (b1/days)) |
| Density (DENSITY) | (b0 + b1 * days) ** ( - 1 / b2) |
| Dhanoa (DHANOA) | b0 * days ** (b1 * days) * Exp( - b2 * days) |
| Dijkstra (DJKSTR) | b0 * Exp(b1 * (1 - Exp( - b2 * days)) / b2) - b3 * days |
| Exponential decline function or Gaines (EDFGAIN) | b0 * Exp( - b1 * days) |
| Gauss (GAUSS) | b0 * (1 - b2 * Exp( - b1 * days ** 2)) |
| Gompertz (GMPRTZ) | b0 * Exp( - b1 * Exp(- b2 * days)) |
| Grossman (GROSMN) | b0 * (days ** b1) * Exp( - b2 * days) * (1 + b3 * SIN(days) + b4 * Cos(days)) |
| Hayashi (HAYSHI) | b1*Exp( - b2/days - Exp(- days / b0 * b2)) |
| Inverse quadratic polynomial (INVQPOL) | days * (b0 + (b1 * days)+ (b2 * (days ** 2))) ** (-1) |
| Inverse, Linear hyperbolic (INVLINHY) | b0 + (b1 / days) |
| Johnson Schumacher (JOHNSCH) | b0 * Exp(- b1 / (days + b2)) |
| Log Logistic (LOGLOG) | b0- ln(1 + b1 * Exp( - b2 * days)) |
| Log Modified Weibull (LGMWEIB) | (b0 +( b2 * days)) ** (b1) |
| Logarithmic (LOGARITH) | b0 + (b1 * ln(days)) |
| Madalena (MADALN) | $b0-b1*days$ |
| Michaelis Menten (MICHMEN) | b1 / days * (1 + (b2 / 210) ** b1) / (1 + (b2 / days) ** b1) * (1 + (days / b2) * b1) |
| MilkBot (MILKBOT) | b0 * (1 - (Exp((b2 * days) / b1)) / 2) * Exp( - b3 * days) |
| Molina and Boschini/Modal Linear (MOL&BOS) | b0 - b1 * Abs(days - (b2)) |
| Morgan Mercer Florin (MORMFLO) | ((b0 * b1) + (b2 * days ** b3)) / (b1 +( days ** b3)) |
| Nelder, inverser polynomial, Yadav (NELDER) | days/(b0+b1*days+b2*days**2) |
| Parabolic exponential and Parabolic, Sikka (PEMSIK) | b0 * Exp((b1 * days) - (b2 * days ** 2)) |
| Parabolic yield-density (PARYLDENS) | (b0 +( b1 * days )+ ((b2 *b2) (days ** 2))) ** (- 1) |
| Power (POWER) | b0 * (days**b1) |
| Quadratic cum log (QDCMLOG) | b0+ b1 * days + b2 * days ** 2 + b3 * ln (days) |
| Quadratic (QUADRT) | b0 + (b1 * days) + (b2 * days**2) |
| Quadratic model Dave (DAVE) | b0 + b1 * days- b2 * days ** 2 |
| Quadratic spline function with one knot (QUADSPL) | b0+ b1 * days + b2 * days ** 2 + b3 * (days - Knot) ** 2 |
| Ratio Cubics/ Partial Fraction with Cubic Denominator (RATCUB) | (b0+b1*days+b2*days**2+b3*days**3)/(b4*days**3) |
| Ratio Quadratics/ Partial Fraction with Quadratic Denominator (RATQUAD) | (b0 + b1 * days + b2 * days ** 2) / (b3 * days ** 2) |
| Richards (RICHRDS) | b0 / ((1 + b2 * Exp(- b1 * days)) ** (1 / b3)) |
| Rook (ROOK) | b0 * (1 / 1 + (b1 / b2 + days)) *Exp(-b3 * days) |
| Simple Linear (SIMLIN) | b0 + (b1 * days) |
| Singh And Gopal (SIN&GOP) | b0-b1*days+b2*ln (days) |
| Third order Legendre orthogonal polynomial (3ORDLEG) | b0 * 0.7071 * (2 * ((days-1)/(210-1))-1) ** 0+ (b1 * $1.2247$ * (2 * ((days-1)/(210-1))-1) ** 1) + ((b2 * $-0.7906$ * (2 * ((days-1)/(210-1))-1) ** 0)+ ($2.3717$ * (2 * ((days-1)/(210-1))-1) ** 2))+ ((b3 * $-2.8062$ * (2 * ((days-1)/(210-1))-1) ** 1)+ ($4.6771$ * (2 * ((days-1)/(210-1))-1) ** 3)) |
| Verhulst/Logistic differential equation/Pearl Reed (VERHLST) | b0 / (1 + b1 * (Exp(- b2 * days))) |
| Von Bertalanffy (VBRTLNFY) | b0 * (1-(b1) *( Exp(-b2 * days))) ** 3 |
| Weibull, Parametric Survival Models (PARSURW) | b0 - (b1 * (Exp(- b2 *( days ** b3)))) |
| Wilmink’s exponential (WILMINK) | b0+b1*Exp(-0.05 * days)+b2 * days |
| Wood (WOOD) | b0 - b1 * Exp(-0.05 * days) - b3 * days |
| Days: days in milk. | |
