# Supplementary material for: Goat Milk Nutritional Quality Software-Automatized Individual Curve Model Fitting, Shape Parameters Calculation and Bayesian Flexibility Criteria Comparison
Source: Animals (Basel). 2020 Sep 18;10(9):1693. doi: 10.3390/ani10091693 (PMC7552780; doi:10.3390/ani10091693)
Supplement: Supplementary file 1 [file animals-10-01693-s001.zip › Table S3.docx]

**Table S3:** Peak yield and persistence estimates across lactation curve models for milk composition and literature references.

| **Model** | **Peak Yield/ change in variable units per event** | **Persistency/ Descending rate of the curve after the lactation Peak/ r(th) relative rate of decline at the point halfway between peak yield and end of lactation/instantaneous rate of change** | **Reference** |
| --- | --- | --- | --- |
| Ali and Schaeffer model (ALISCH) | $b_{0}$ | $P_{2:1}=$Milk yield in the second 100 DIM divided by yield in the first 100 DIM.  $P_{3:1}=$Milk yield in the third 100 DIM divided by yield in the first 100 DIM.  $P_{d}=$Milk yield at 280 DIM divided by milk yield at 60 DIM.  All three measures were expressed as percentages. | Ali and Schaeffer,1987; Otwinowska-Mindur, 2015 and Strucken, 2011 |
| Asymptotic Regression, Single Exponential decay to an arbitrary value (SXPDCY) | ΔN/N  (the peak measures the change in variable units per event) | R = -ΔN/Δt  ΔN= increase in the number of units | Garson, 2013 and Urbina, 2018 |
| Asymptotic Regression,Lactation modification of Metcherlich Law of Diminishing Returns or Exponential growth model (METLAW) | $b_{0}-\frac{b_{3}\left[ 1+ln\left( \frac{b_{0}b_{1}b_{2}}{b_{2}} \right) \right]}{b_{2}}$ | R = ΔN/Δt | Prasad, 2007 |
| Brody (BRODY) | $\left( b_{2}- b_{1} \right)^{-1}ln\left( \frac{b_{2}}{b_{1}} \right)$ | ${b_{0}e}^{{-b}_{2}Days}\left[ \left( b_{1}+b_{2} \right)Exp\left( -b_{1}Days \right)-b_{2} \right]$ | Brody et al, 1923; Sherchand, 1995; Duque, 2018 and Pulina, 2005 |
| Cappio Borlino, biexponential (CAPBOR) | Empirically obtained by finding the largest value among daily yields predicted by the model for the whole lactation interval.  $b_{2}= \frac{1}{\left( t_{m}log t_{m} \right)}$  $t_{m}=e^{\left( \frac{1}{b_{2}t_{m}} \right)}$  $t_{m}$ is time at peak yield and is calculate iteratively. | $\frac{b_{0}}{b_{1}b_{2}}$ | Cappio-Borlino et al., 1995; Martinez, 2018; Franci, 1999 and Gipson, 1990 |
| Cobby and Le Du (COBLDU) | $\left( \frac{b_{0}-b_{2}}{b_{1}} \right)-{b_{2}b_{1}}^{-1}ln\left( \frac{b_{0}b_{1}}{b_{2}} \right)$ | ${b_{0}b_{1}e}^{{-b}_{1}Days}-b_{2}$ | Cobby and Le Du, 1978 and Duque, 2018 |
| Compound/ Exponential Growth (CEXPGR) | Peak=ΔN/N | $R= b_{1}-1$ | Hobbie, 2007; Roberts, 2020 and Urbina, 2018 |
| Cubic (CUBIC) | $b_{0}+b_{1}\left( -b_{2}-\frac{\sqrt{b_{2}^{2}-3b_{1}b_{3}}}{3b_{3}} \right)+b_{2}\left( -b_{2}-\frac{\sqrt{b_{2}^{2}-3b_{1}b_{3}}}{3b_{3}} \right)^{2}+b_{3}\left( -b_{2}-\frac{\sqrt{b_{2}^{2}-3b_{1}b_{3}}}{3b_{3}} \right)^{3}$ | $\left[ 1-\left( \frac{y_{pm-}y_{lm}}{y_{pm}} \right) \right]*100$  Where Ypm, average daily milk yield of peak month; Ylm, average daily milk yield of 10th or 12th or 14th or 16th month of lactation. | Mehta, 2015  Keskin, et al. 2006 |
| Cubic Spline function with one knot (CUBSPL) | $\sqrt{3b_{3}x^{2}+2b_{2}x+3b_{4}\left( Days-Knot \right)^{2}{+b}_{1}}$ | To calculate and analyse the Persistency or rates of descent (RD) in their different phases, such as: initial persistency (IP), final persistency (FP) and total persistency (TP). Where IP: defined as the difference between the area under the LC in the middle third (71–140 days) and initial from 11–70 days. FP: defined as the difference between the area under the LC in the final third (141–210 days) and initial third of 11–70 days. TP: defined as the difference between the accumulated production of days 71–210 and the average of the initial third of 11–70 days. The P obtained is dimensionless. | [Verbyla & Verbyla, 2009](#_ENREF_40); [Cankaya et al., 2014](#_ENREF_8) and [Boyd, 2006](#_ENREF_7) |
| Curve S (CURVES) | ΔN/N  (the peak measures the change in variable units per event)  Effectively exponential functions increase throughout their domain (the actual line) since their derivative (themselves) are positive throughout the actual line but as any other continuous function reaches a minimum and a maximum if we consider a closed interval (theorem of Weierestrass) | R = ΔN/Δt  ΔN= increase in the number of units | Sikka, 1950; Mohanty, 2017; Biswal, 2017; Kokate, 2019; Cankaya, 2011; Zadeh, 2019 ;Brody et al, 1923;Bouallegue, 2019 and Korkmaz, 2011 |
| Density (DENSITY) | $-\frac{b_{1}\left( b_{0}+b_{1}Days \right)^{\frac{-1-b_{2}}{b_{2}}}}{b_{2}}$ | R = ΔN/Δt  ΔN= increase in the number of units | [Mellado et al., 2011](#_ENREF_26); [Nguyen et al., 2019](#_ENREF_29) and [Webster, 2018](#_ENREF_41) |
| Dhanoa (DHANOA) | ${b_{0}b_{1}}^{b_{1}b_{2}}e^{b_{1}b_{2}}$ | $-\left( b_{1}b_{2}+1 \right)ln\left( b_{2} \right)$ | Dhanoa and Le Du,1982 and Korkmaz, 2011 |
| Dijkstra (DJKSTR) | $b_{2}^{-1}ln\left( \frac{b_{1}}{b_{3}} \right)$ | $b_{1}e\left( {-b}_{2}\left( \frac{\left( b_{2}^{-1}ln\left( \frac{b_{1}}{b_{3}} \right) \right)+lactation length in days}{2} \right) \right)-b_{3}$ | Dijkstra et al., 1997 and Nasri, 2008 |
| Exponential decline function or Gaines (EDFGAIN) | ΔN/N  (the peak measures the change in variable units per event) | R = -ΔN/Δt  ΔN= increase in the number of units | Brody et al, 1923 and Bouallegue, 2019 and Korkmaz, 2011 |
| Gauss (GAUSS) | $b_{0}$ | $\sqrt{\sigma}=SD$ | Guo, 2011 |
| Gompertz (GMPRTZ) | $b_{0}b_{2}e^{\left( \frac{b_{1}}{b_{2}}-1 \right)}$ | $b_{1}e^{\left( -b_{2}\left( \frac{\frac{ln\left( b_{2} \right)}{b_{1}}+Total Days of Lactation}{2} \right) \right)}-b_{2}$ | Bahashwan, 2018 and Nasri, 2008 |
| Grossman (GROSMN) | $b_{0}b_{1}$ | $-\left( 1+b_{1} \right)ln\left( b_{2} \right)$  $b_{2}^{{-b}_{1}+1}$ | Grossman et al., 1986 and Soysal, 2004 |
| Hayashi (HAYSHI) | $b_{1}\left( {b_{0}}^{\left( \frac{b_{0}}{1-b_{0}} \right)}- {b_{0}}^{\left( \frac{1}{1- b_{0}} \right)} \right)$ | $b_{2}$  The parameter “$b_{2}$" concerns lactation length and determines the declining slope of yield after the day of maximum yield. | Hayashi et al, 1986; [林孝, 1993](#_ENREF_45) and [Korkmaz et al., 2011](#_ENREF_20) |
| Inverse quadratic polynomial (INVQPOL) | $\frac{1}{2\sqrt{b_{1}b_{2}+b_{0}}}$ | $b_{2}$  describes the post peak decline. | Nelder, 1966; [Biswal et al., 2017](#_ENREF_5) and [Olori, 1997](#_ENREF_31) |
| Inverse, linear Hyperbolic.(INVLINHY) | $b_{0}+2\sqrt{b_{1}b_{2}}$ | $b_{1}$ | [Faro & Albuquerque, 2002](#_ENREF_13) |
| Johnson Schumacher (JOHNSCH) | $\frac{4b_{0}}{b_{1}^{2}b_{2}} e^{b_{1}b_{2}-2}$ | $\left( b_{1} \right)$ | [Ghavi Hossein-Zadeh, 2017](#_ENREF_16) |
| Log Logistic (LOGLOG) | $b_{1}$ | $b_{2}$ | [Pina Pérez et al., 2007](#_ENREF_33) and [Bebbington et al., 2009b](#_ENREF_4) |
| Log Modified Weibull (LGMWEIB) | $b_{1}$ | $b_{2}$ | [Carrasco et al., 2008](#_ENREF_9) |
| Logarithmic (LOGARITH) | $b_{1}$ | $b_{2}$ | [Dongre & Gandhi, 2013](#_ENREF_11) |
| Madalena (MADALN) | $b_{1}=\frac{\Delta y}{\Delta Days}$ | To get an indication of lactation persistence, the 305-day lactation yield was divided by peak yield ${(b}_{1})$to give peak yield factors for each group of animals.  $\frac{Total Milk Yield}{b_{1}}$ | Quinn, 2005; López, 2015 [Fresno Baquero et al., 1992](#_ENREF_15) and [Madalena et al., 1979](#_ENREF_24) |
| Michaelis Menten (MICHMEN) | $\frac{b_{1}+1}{4b_{1}\left( b_{2}\left[ \frac{\left( b_{1} 1 \right)}{\left( b_{1}+1 \right)} \right]^{\frac{1}{b_{1}}} \right)}\left[ \left( b_{1} 1 \right)+\left( b_{1}+1 \right)\left( \frac{\left( b_{2}\left[ \frac{\left( b_{1} 1 \right)}{\left( b_{1}+1 \right)} \right]^{\frac{1}{b_{1}}} \right)}{210} \right)^{b_{1}} \right]$ | $b_{2}$ | Rook et al., 1993 and [Rebouças et al., 2008](#_ENREF_34) |
| MilkBot (MILKBOT) | $-b_{1}\ln\left( \frac{2 b_{3}b_{1}}{b_{3}b_{1}+1} \right){+b}_{2}$ | $\frac{0.693}{b_{3}}$ | [Ehrlich, 2011](#_ENREF_12) |
| Molina and Boschini/Modal Linear (MOL&BOS) | $b_{0}$ | $100\left[ 1-\frac{b_{1}}{b_{0}}\frac{\left( Lactation days+b_{2} \right)}{2} \right]$  or  $\frac{Total Milk Yield}{b_{0}}100$  $b_{2}=Peak day$ | Quinn, 2005 and [Molina & Boschini, 1979](#_ENREF_28) |
| Morgan Mercer Florin (MORMFLO) | $\frac{\left( 1+\frac{1}{b_{3}} \right)b_{0}+\left( 1-\frac{1}{b_{3}} \right)b_{2}}{2}$ | $\frac{Total Milk yield-Peak}{Total Lactation Days-\left( \sqrt[b_{3}]{b_{1}}\left( \frac{b_{3}+1}{b_{3}-1} \right)^{\frac{1}{b_{3}}} \right)}$ | López, 2015 |
| Nelder, inverser polynomial, Yadav (NELDER) | $\frac{1}{b_{1}+2\sqrt{b_{0}b_{2}}}$ | $\frac{\left( b_{0}-b_{2}{Days}^{2} \right)}{\left( b_{0}+b_{1}Days+b_{2}Days+b_{2}{Days}^{2} \right)^{2}}$ | Nanda, 2019 and Duque, 2018 |
| Parabolic exponential model and Parabolic, Sikka (PEMSIK) | $b_{0}e^{\left( \frac{b_{1}^{2}}{4b_{2}} \right)}$ | $\sum_{i=1}^{k} \frac{\left( P_{i+1} \right)/P_{i}}{k}*100$  Where pi  is the yield of the record i that starts at peak time and k   is the record number from peak time to end of lactation. | Sikka, 1950; Mohanty, 2017; Biswal, 2017; Kokate, 2019 ;Cankaya, 2011 and Zadeh, 2019 |
| Parabolic yield-density (PARYLDENS) | $\frac{-b_{1}}{{2b}_{2}}$ | $2b_{0}Days+b_{1}$ | [Yahuza, 2011](#_ENREF_42) |
| Power (POWER) | $b_{0}\left( b_{1} \right){Days}^{b_{1}-1}$ | $b_{1}$ | [Mellado et al., 2011](#_ENREF_26); [Nguyen et al., 2019](#_ENREF_29) and [Webster, 2018](#_ENREF_41) |
| Quadratic cum log model (QDCMLOG) | $\frac{b_{2}}{b_{1}}$ | $r=\frac{ln\left( \Delta y \right)}{\Delta Days}$ | Malhotra et al., 1980; Biswal, 2017; [Lombaard, 2006](#_ENREF_23) and [Gupta et al., 2016](#_ENREF_17) |
| Quadratic model (QUADRT) | $b_{0}+b_{1}\left( \frac{-b_{1}}{2b_{2}} \right)+b_{2}\left( \frac{-b_{1}}{2b_{2}} \right)^{2}$ | $\frac{1}{b_{2}}$ | Biswal, 2017 and [Noguera et al., 2011](#_ENREF_30) |
| Quadratic model Dave (DAVE) | $b_{0}\left( \frac{b_{1}}{b_{2}} \right)e\left( {-b}_{2} \right)$ | $-\left( b_{1}+1 \right)ln\left( b_{2} \right)$ | Dave, 1971; [Bangar & Verma, 2017](#_ENREF_2) and Martínez, 2018 |
| Quadratic spline function with one knot (QUADSPL) | $\sqrt{2b_{2}x+2b_{3}\left( Days-Knot \right)^{2}{+b}_{1}}$ | To calculate and analyse the Persistency or rates of descent (RD) in their different phases, such as: initial persistency (IP), final persistency (FP) and total persistency (TP). Where IP: defined as the difference between the area under the LC in the middle third (71–140 days) and initial from 11–70 days. FP: defined as the difference between the area under the LC in the final third (141–210 days) and initial third of 11–70 days. TP: defined as the difference between the accumulated production of days 71–210 and the average of the initial third of 11–70 days. The P obtained is dimensionless. | [Verbyla & Verbyla, 2009](#_ENREF_40); [Cankaya et al., 2014](#_ENREF_8) and [Boyd, 2006](#_ENREF_7) |
| Ratio Cubics/ Partial Fraction with Cubic Denominator (RATCUB) | $\frac{-b_{2}{Days}^{2}-{2b}_{1}Days-3b_{0}}{b_{4}{Days}^{4}}$ | R = ΔN/Δt  ΔN= increase in the number of units | [Kung, 2006](#_ENREF_21) |
| Ratio Quadratics/ Partial Fraction with Quadratic Denominator (RATQUAD) | $\frac{-b_{1}Days-2b_{0}}{b_{3}{Days}^{3}}$ | R = ΔN/Δt  ΔN= increase in the number of units | [Kung, 2006](#_ENREF_21) |
| Richards (RICHRDS) | $\sqrt[b_{3}]{b_{2}+1}\left( \frac{1}{b_{3}+1} \right)^{\frac{1}{b_{3}}}$ | $\frac{Total Milk yield-Peak}{Total Lactation Days-\left( \frac{1}{b_{1}}ln\left( \frac{b_{2}}{b_{3}} \right) \right)}$ | Richards,1959; López, 2015 and Bayram, 2004 |
| Rook (ROOK) | $\frac{b_{0}e^{{-b}_{2}\left( -\left( \frac{b}{2}+b_{2} \right)+\sqrt{\left( \left( \frac{b_{1}}{2}+b_{2} \right)^{2}-b_{2}\left( b_{1}+b_{2} \right)+\frac{b_{1}}{b_{3}} \right)} \right)}}{\left( 1+\frac{b_{1}}{\left( b_{2}+ -\left( \frac{b_{1}}{2}+b_{2} \right)+\sqrt{\left( \left( \frac{b_{1}}{2}+b_{2} \right)^{2}-b_{2}\left( b_{1}+b_{2} \right)+\frac{b_{1}}{b_{3}} \right)} \right)} \right)}$ | The persistency measures used in this study were ratios between different parts of the lactation (P2:1, P3:1 and PWeller). The P2:1 and P3:1 were proposed by Johansson and Hansson (1940). P2:1 and P3:1 are the ratios between the MYs of the second and third 100 days of lactation, respectively, and that of the ﬁrst 100 days. Also, Weller et al. (2006) de ﬁned milk persistency as estimated milk production at 180 day after peak divided by estimated peak production in percent as follows:  $P_{Weller}=100 \frac{PROD \left( 270 \right)}{PROD \left( 90 \right)}$  where PROD(270) and PROD(90) are milk production at 270 and 90 days in milk, respectively. | [Zadeh, 2019](#_ENREF_43) |
| Simple Linear (SIMLIN) | $b_{1}=\frac{\Delta y}{\Delta Days}$ | To get an indication of lactation persistence, the 305-day lactation yield was divided by peak yield ${(b}_{1})$to give peak yield factors for each group of animals.  $\frac{Total Milk Yield}{b_{1}}$ | [Marsh, 1982](#_ENREF_25) and [Singh & Kumar, 2007](#_ENREF_38) |
| Singh And Gopal (SIN&GOP) | $\frac{b_{2}}{b_{1}}$ | $\frac{1}{b_{2}}$ | Quinn, 2005; [Sherchand et al., 1995](#_ENREF_36); [Singh & Gopal, 1982](#_ENREF_39); [Bouallegue & M’Hamdi, 2019](#_ENREF_6) and [Noguera et al., 2011](#_ENREF_30) |
| Third order Legendre ortogonal polynomial (3ORDLEG) | $\sqrt{\begin{aligned} 0.01171b_{1}+0.00021\left( 2Days-211 \right)-0.02685b_{3}+ \\ 0.13427\left( 0.00956 Days-1.00956 \right) \end{aligned}}$ | to calculate and analyse the Persistency or rates of descent (RD) in their different phases, such as: initial persistency (IP), final persistency (FP) and total persistency (TP). Where IP: defined as the difference between the area under the LC in the middle third (71–140 days) and initial from 11–70 days. FP: defined as the difference between the area under the LC in the final third (141–210 days) and initial third of 11–70 days. TP: defined as the difference between the accumulated production of days 71–210 and the average of the initial third of 11–70 days. The P obtained is dimensionless. | [Schaeffer, 2004](#_ENREF_35); [León et al., 2012](#_ENREF_22) and [Boyd, 2006](#_ENREF_7) |
| Verhulst/Logistic differential equation/Pearl Reed (VERHLST) | $\frac{b_{2}}{2}$ | $r=\frac{ln\left( \Delta y \right)}{\Delta Days}$ | [Herman & Strang, 2016](#_ENREF_18) and [Pearl & Reed, 1920](#_ENREF_32) |
| Von Bertalanffy (VBRTLNFY) | $\frac{b_{1}}{b_{0}}$  corresponds to the ratio between the intercept and the slope of the regression line | The persistency measures used in this study were ratios betwen different parts of the lactation (P_2:1_, P_3:1_ and P_Weller_). The P2:1 and P3:1 were proposed by Johansson and Hansson (1940). P2:1 and P3:1 are the ratios between the MYs of the second and third 100 days of lactation, respectively, and that of the ﬁrst 100 days. Also, Weller et al. (2006) de ﬁned milk persistency as estimated milk production at 180 day after peak divided by estimated peak production in percent as follows:  $P_{Weller}=100 \frac{PROD \left( 270 \right)}{PROD \left( 90 \right)}$  where PROD(270) and PROD(90) are milk production at 270 and 90 days in milk, respectively. | Bahashwan, 2018;[Charruau, 2011](#_ENREF_10) and [Johansson & Hansson, 1940](#_ENREF_19) |
| Weibull, Parametric Survival Models (PARSURW) | $b_{0}e\left( -0.5 \left( \frac{ln\left( \frac{Days}{b_{1}} \right)}{b_{2}} \right)2 \right)$  $b_{2}\left( \frac{k-1}{k} \right)^{\frac{1}{k}}$  $k=b_{1}$ | $k=b_{1}$  $b_{2}=Exp \left( \frac{b_{0}}{b_{1}} \right)$ | Bebbington, 2009; [ZunZunSite3, 2020](#_ENREF_44); [Bebbington et al., 2009a](#_ENREF_3) and [Franco García, 2016](#_ENREF_14) |
| Wilmink’s exponential (WILMINK) | $b_{0}+b_{1}\left( -20\left( ln20 \frac{b_{0}}{b_{2}} \right)+b_{2}^{e-0.05-20\left( ln20 \frac{b_{1}}{b_{2}} \right)} \right)$ | $b_{1}$  regression coefficient related to production decrease after peak yield (persistency) | Wilmink, 1987; [Silvestre et al., 2006](#_ENREF_37); [Mikhchi et al., 2015](#_ENREF_27) and [Arslan et al., 2004](#_ENREF_1) |
| Wood (WOOD) | $\frac{b_{0}}{\left( \frac{b_{2}}{b_{1}} \right)^{b_{1}}e^{b_{1}}}$ | $-\left( b_{1}+1 \right)lnb_{2}$ | Wood, 1967 and Nasri, 2008; Gipson, 1990 and Bordonaro, 2013 |
