# Supplementary material for: Goat Milk Nutritional Quality Software-Automatized Individual Curve Model Fitting, Shape Parameters Calculation and Bayesian Flexibility Criteria Comparison
Source: Animals (Basel). 2020 Sep 18;10(9):1693. doi: 10.3390/ani10091693 (PMC7552780; doi:10.3390/ani10091693)
Supplement: Supplementary file 1 [file animals-10-01693-s001.zip › Table S4.docx]

**Table S4:** Summary of descriptive statistics for milk protein, fat, dry matter and lactose content (%) and somatic cells count (sc/mL) in the Murciano-Granadina goat breed (N=3107 records for each milk component).

| Descriptive statistics | Protein | Fat | Dry Matter | Lactose | Somatic Cells Count |
| --- | --- | --- | --- | --- | --- |
| Mean | 3.542 | 5.330 | 14.457 | 4.809 | 805.677 |
| Std. Error of Mean (SEM) | 0.009 | 0.021 | 0.026 | 0.006 | 21.494 |
| Median | 3.470 | 5.270 | 14.360 | 4.820 | 399.000 |
| Std. Deviation (SD) | 0.501 | 1.155 | 1.442 | 0.316 | 1198.094 |
| Coefficient of variation (CV), % | 0.141 | 0.217 | 0.100 | 0.066 | 1.487 |
| Variance | 0.251 | 1.334 | 2.081 | 0.100 | 1435429.403 |
| Skewness | 2.138 | 0.673 | 0.563 | -0.813 | 3.959 |
| Std. Error of Skewness | 0.044 | 0.044 | 0.044 | 0.044 | 0.044 |
| Kurtosis | 10.779 | 1.657 | 1.201 | 7.487 | 20.330 |
| Std. Error of Kurtosis | 0.088 | 0.088 | 0.088 | 0.088 | 0.088 |
| Minimum | 2.530 | 2.390 | 11.090 | 2.130 | 12.000 |
| Maximum | 7.480 | 11.830 | 22.140 | 6.130 | 9756.000 |
| Quartile coefficient of dispersion | 0.077 | 0.142 | 0.066 | 0.037 | 0.679 |
