# Supplementary material for: Goat Milk Nutritional Quality Software-Automatized Individual Curve Model Fitting, Shape Parameters Calculation and Bayesian Flexibility Criteria Comparison
Source: Animals (Basel). 2020 Sep 18;10(9):1693. doi: 10.3390/ani10091693 (PMC7552780; doi:10.3390/ani10091693)
Supplement: Supplementary file 1 [file animals-10-01693-s001.zip › Table S5.docx]

**Table S4:** Mean and standard deviation (SD) for individual adjusted coefficient of determination (Adj. R^2^) of the models of curves for milk protein (%), fat (%), dry matter (%), lactose (%) and somatic cells count (sc/mL) in Murciano-Granadina goats.

| **Model** | **Protein**  **(%)** | | **Fat**  **(%)** | | **Dry Matter**  **(%)** | | **Lactose**  **(%)** | | **Somatic cells count**  **(sc/mL)** | |
| --- | --- | --- | --- | --- | --- | --- | --- | --- | --- | --- |
|  | **Adj. R^2^ Mean** | **SD** | **Adj. R^2^ Mean** | **SD** | **Adj. R^2^ Mean** | **SD** | **Adj. R^2^ Mean** | **SD** | **Adj. R^2^ Mean** | **SD** |
| Ali and Schaeffer model (ALISCH) | 0.548 | 0.239 | 0.374 | 0.228 | 0.429 | 0.245 | 0.624 | 0.217 | 0.401 | 0.231 |
| Asymptotic Regression, Single Exponential decay to an arbitrary value (SXPDCY) | 0.241 | 0.214 | 0.156 | 0.175 | 0.173 | 0.187 | 0.386 | 0.259 | 0.222 | 0.241 |
| Asymptotic Regression, Lactation modification of Metcherlich Law of Diminishing Returns or Exponential growth model (METLAW) | 0.439 | 0.260 | 0.289 | 0.237 | 0.334 | 0.259 | 0.526 | 0.261 | 0.397 | 0.256 |
| Brody (BRODY) | 0.255 | 0.219 | 0.182 | 0.178 | 0.191 | 0.193 | 0.424 | 0.252 | 0.251 | 0.256 |
| Cappio Borlino, biexponential (CAPBOR) | 0.432 | 0.237 | 0.278 | 0.216 | 0.325 | 0.240 | 0.530 | 0.234 | 0.333 | 0.238 |
| Cobby and Le Du (COBLDU) | 0.286 | 0.243 | 0.176 | 0.172 | 0.187 | 0.186 | 0.439 | 0.249 | 0.280 | 0.182 |
| Compound/ Exponential Growth (CEXPGR) | 0.047 | 0.482 | 0.003 | 0.026 | 0.006 | 0.062 | 0.057 | 0.579 | 0.267 | 0.267 |
| Cubic (CUBIC) | 0.522 | 0.239 | 0.349 | 0.236 | 0.407 | 0.253 | 0.609 | 0.222 | 0.379 | 0.231 |
| Cubic Spline function with one knot (CUBSPL) | 0.522 | 0.239 | 0.349 | 0.236 | 0.407 | 0.253 | 0.609 | 0.222 | 0.379 | 0.231 |
| Curve S (CURVES) | 0.205 | 0.200 | 0.123 | 0.151 | 0.155 | 0.185 | 0.299 | 0.206 | 0.215 | 0.212 |
| Density (DENSITY) | NC | NC | NC | NC | NC | NC | NC | NC | NC | NC |
| Dhanoa (DHANOA) | 0.451 | 0.241 | 0.280 | 0.223 | 0.341 | 0.254 | 0.539 | 0.241 | 0.354 | 0.247 |
| Dijkstra (DJKSTR) | 0.519 | 0.241 | 0.338 | 0.242 | 0.384 | 0.257 | 0.583 | 0.222 | 0.364 | 0.241 |
| Exponential decline function or Gaines (EDFGAIN) | 0.241 | 0.214 | 0.156 | 0.175 | 0.173 | 0.187 | 0.386 | 0.259 | 0.250 | 0.236 |
| Gauss (GAUSS) | NC | NC | NC | NC | NC | NC | NC | NC | NC | NC |
| Gompertz (GMPRTZ) | 0.003 | 0.028 | 0.002 | 0.053 | 0.003 | 0.120 | NC | NC | NC | NC |
| Grossman (GROSMN) | 0.494 | 0.220 | 0.364 | 0.220 | 0.407 | 0.235 | 0.584 | 0.217 | 0.451 | 0.250 |
| Hayashi (HAYSHI) | 0.260 | 0.215 | 0.157 | 0.164 | 0.185 | 0.198 | 0.343 | 0.229 | 0.237 | 0.217 |
| Inverse quadratic polynomial (INVQPOL) | NC | NC | NC | 0.531 | NC | NC | NC | NC | NC | NC |
| Inverse, linear Hyperbolic (INVLINHY) | 0.204 | 0.200 | 0.125 | 0.155 | 0.157 | 0.189 | 0.304 | 0.209 | 0.141 | 0.170 |
| Johnson Schumacher (JOHNSCH) | 0.299 | 0.231 | 0.200 | 0.194 | 0.259 | 0.229 | 0.434 | 0.268 | 0.199 | 0.231 |
| Log Logistic (LOGLOG) | NC | NC | NC | NC | 0.002 | 0.102 | NC | NC | NC | NC |
| Log Modified Weibull (LGMWEIB) | NC | NC | NC | NC | NC | NC | 0.016 | 0.794 | NC | NC |
| Logarithmic (LOGARITH) | 0.256 | 0.224 | 0.152 | 0.173 | 0.187 | 0.205 | 0.414 | 0.253 | 0.183 | 0.178 |
| Madalena (MADALN) | 0.238 | 0.211 | 0.152 | 0.171 | 0.170 | 0.184 | 0.170 | 0.257 | 0.187 | 0.181 |
| Michaelis Menten (MICHMEN) | NC | NC | NC | NC | NC | NC | NC | NC | NC | NC |
| MilkBot (MILKBOT) | 0.295 | 0.246 | 0.146 | 0.180 | 0.200 | 0.199 | 0.430 | 0.251 | 0.313 | 0.274 |
| Molina and Boschini/Modal Linear (MOL&BOS) | 0.339 | 0.226 | 0.221 | 0.207 | 0.226 | 0.201 | 0.456 | 0.252 | 0.188 | 0.183 |
| Morgan Mercer Florin (MORMFLO) | 0.445 | 0.276 | 0.254 | 0.206 | 0.097 | 0.291 | 0.524 | 0.259 | NC | NC |
| Nelder, inverser polynomial, Yadav (NELDER) | NC | NC | NC | NC | NC | NC | NC | NC | NC | NC |
| Parabolic exponential model and Parabolic, Sikka (PEMSIK) | 0.433 | 0.245 | 0.275 | 0.222 | 0.327 | 0.248 | 0.539 | 0.249 | 0.353 | 0.254 |
| Parabolic yield-density (PARYLDENS) | NC | NC | NC | NC | NC | NC | NC | NC | 0.481 | 0.310 |
| Power (POWER) | 0.261 | 0.227 | 0.155 | 0.173 | 0.189 | 0.205 | 0.411 | 0.251 | 0.233 | 0.232 |
| Quadratic cum log model (QDCMLOG) | 0.509 | 0.234 | 0.341 | 0.230 | 0.394 | 0.247 | 0.598 | 0.226 | 0.363 | 0.233 |
| Quadratic model (QUADRT) | 0.430 | 0.243 | 0.275 | 0.223 | 0.327 | 0.248 | 0.540 | 0.250 | 0.305 | 0.228 |
| Quadratic model Dave (DAVE) | 0.430 | 0.243 | 0.275 | 0.223 | 0.327 | 0.248 | 0.540 | 0.250 | 0.305 | 0.228 |
| Quadratic spline function with one knot (QUADSPL) | 0.430 | 0.243 | 0.275 | 0.223 | 0.327 | 0.248 | 0.540 | 0.250 | 0.305 | 0.228 |
| Ratio Cubics/ Partial Fraction with Cubic Denominator (RATCUB) | 0.422 | 0.250 | 0.278 | 0.213 | 0.321 | 0.243 | 0.534 | 0.239 | 0.294 | 0.221 |
| Ratio Quadratics/ Partial Fraction with Quadratic Denominator (RATQUAD) | 0.318 | 0.231 | 0.200 | 0.186 | 0.239 | 0.223 | 0.452 | 0.242 | 0.217 | 0.196 |
| Richards (RICHRDS) | 0.013 | 0.208 | 0.029 | 0.243 | 0.002 | 0.170 | 0.037 | 0.098 | 0.000 | 0.000 |
| Rook (ROOK) | 0.302 | 0.247 | 0.186 | 0.182 | 0.302 | 0.202 | 0.439 | 0.251 | 0.272 | 0.231 |
| Simple Linear (SIMLIN) | 0.238 | 0.211 | 0.152 | 0.171 | 0.170 | 0.184 | 0.382 | 0.257 | 0.187 | 0.181 |
| Singh And Gopal (SIN&GOP) | 0.434 | 0.237 | 0.279 | 0.218 | 0.327 | 0.242 | 0.531 | 0.235 | 0.304 | 0.230 |
| Third order Legendre ortogonal polynomial (3ORDLEG) | NC | NC | 0.159 | 0.061 | 0.400 | 0.195 | NC | NC | 0.195 | 0.182 |
| Verhulst/Logistic differential equation/Pearl Reed (VERHLST) | 0.303 | 0.273 | 0.179 | 0.207 | 0.230 | 0.254 | 0.448 | 0.281 | 0.000 | NC |
| Von Bertalanffy (VBRTLNFY) | 0.314 | 0.263 | 0.204 | 0.212 | 0.233 | 0.244 | 0.461 | 0.282 | NC | NC |
| Weibull, Parametric Survival Models (PARSURW) | NC | 0.000 | NC | 0.000 | NC | 0.000 | NC | NC | 0.000 | NC |
| Wilmink’s exponential (WILMINK) | 0.429 | 0.238 | 0.277 | 0.214 | 0.322 | 0.239 | 0.523 | 0.228 | 0.299 | 0.227 |
| Wood (WOOD) | 0.429 | 0.238 | 0.277 | 0.214 | 0.322 | 0.239 | 0.523 | 0.228 | 0.299 | 0.227 |
|  |  | |  | |  | | NC: Model did not converge. | |  | |
