# Supplementary material for: Goat Milk Nutritional Quality Software-Automatized Individual Curve Model Fitting, Shape Parameters Calculation and Bayesian Flexibility Criteria Comparison
Source: Animals (Basel). 2020 Sep 18;10(9):1693. doi: 10.3390/ani10091693 (PMC7552780; doi:10.3390/ani10091693)
Supplement: Supplementary file 1 [file animals-10-01693-s001.zip › Table S6.docx]

**Table S6:** Bayes factor inference of Pearson’s pairwise correlations among protein, fat, dry matter, lactose (%) and somatic cells count (sc/mL) estimates of curve shape parameters.

| **Parameters** | | | **b_0_** | **b_1_** | **b_2_** | **b_3_** | **b_4_** |
| --- | --- | --- | --- | --- | --- | --- | --- |
| Protein (%) | b_0_ | Pearson Correlation | 1 | -0.022 | 0.004 | -0.14 | 0.459 |
|  |  | Bayes Factor |  | 8.584 | 7.717 | 4.636 | 2.294 |
|  | b_1_ | Pearson Correlation | -0.022 | 1 | -0.011 | 0.125 | -0.353 |
|  |  | Bayes Factor | 8.584 |  | 7.703 | 4.76 | 2.566 |
|  | b_2_ | Pearson Correlation | 0.004 | -0.011 | 1 | -0.112 | 0.378 |
|  |  | Bayes Factor | 7.717 | 7.703 |  | 4.749 | 2.509 |
|  | b_3_ | Pearson Correlation | -0.14 | 0.125 | -0.112 | 1 | 0.996 |
|  |  | Bayes Factor | 4.636 | 4.76 | 4.749 |  | 0.065 |
|  | b_4_ | Pearson Correlation | 0.459 | -0.353 | 0.378 | 0.996 | 1 |
|  |  | Bayes Factor | 2.294 | 2.566 | 2.509 | 0.065 |  |
| Fat (%) | b_0_ | Pearson Correlation | 1 | 0.005 | 0.001 | -0.075 | -0.299 |
|  |  | Bayes Factor |  | 8.674 | 7.718 | 5.097 | 2.680 |
|  | b_1_ | Pearson Correlation | 0.005 | 1 | 0.364 | -0.014 | 0.293 |
|  |  | Bayes Factor | 8.674 |  | 0.724 | 5.289 | 2.691 |
|  | b_2_ | Pearson Correlation | 0.001 | 0.364 | 1 | -0.533 | -0.101 |
|  |  | Bayes Factor | 7.718 | 0.724 |  | 0.640 | 2.940 |
|  | b_3_ | Pearson Correlation | -0.075 | -0.014 | -0.533 | 1 | 0.991 |
|  |  | Bayes Factor | 5.097 | 5.289 | 0.640 |  | 0.106 |
|  | b_4_ | Pearson Correlation | -0.299 | 0.293 | -0.101 | 0.991 | 1 |
|  |  | Bayes Factor | 2.680 | 2.691 | 2.940 | 0.106 |  |
| Dry matter (%) | b_0_ | Pearson Correlation | 1 | 0.080 | 0.018 | -0.094 | -0.398 |
|  |  | Bayes Factor |  | 7.607 | 7.778 | 4.987 | 2.459 |
|  | b_1_ | Pearson Correlation | 0.080 | 1 | 0.304 | -0.016 | -0.692 |
|  |  | Bayes Factor | 7.607 |  | 1.493 | 5.287 | 1.486 |
|  | b_2_ | Pearson Correlation | 0.018 | 0.304 | 1 | -0.405 | 0.719 |
|  |  | Bayes Factor | 7.778 | 1.493 |  | 1.675 | 1.377 |
|  | b_3_ | Pearson Correlation | -0.094 | -0.016 | -0.405 | 1 | 0.778 |
|  |  | Bayes Factor | 4.987 | 5.287 | 1.675 |  | 1.131 |
|  | b_4_ | Pearson Correlation | -0.398 | -0.692 | 0.719 | 0.778 | 1 |
|  |  | Bayes Factor | 2.459 | 1.486 | 1.377 | 1.131 |  |
| Lactose (%) | b_0_ | Pearson Correlation | 1 | -0.002 | -0.019 | 0.363 | 0.146 |
|  |  | Bayes Factor |  | 8.767 | 7.771 | 2.040 | 2.904 |
|  | b_1_ | Pearson Correlation | -0.002 | 1 | -0.127 | 0.447 | -0.423 |
|  |  | Bayes Factor | 8.767 |  | 5.919 | 1.176 | 2.393 |
|  | b_2_ | Pearson Correlation | -0.019 | -0.127 | 1 | -0.928 | 0.926 |
|  |  | Bayes Factor | 7.771 | 5.919 |  | 0.000 | 0.460 |
|  | b_3_ | Pearson Correlation | 0.363 | 0.447 | -0.928 | 1 | 0.998 |
|  |  | Bayes Factor | 2.040 | 1.176 | 0.000 |  | 0.046 |
|  | b_4_ | Pearson Correlation | 0.146 | -0.423 | 0.926 | 0.998 | 1 |
|  |  | Bayes Factor | 2.904 | 2.393 | 0.460 | 0.046 |  |
| Somatic cells count (sc/mL) | b_0_ | Pearson Correlation | 1 | 0.867 | 0.008 | 0.02 | 0.216 |
|  |  | Bayes Factor |  | 0.000 | 7.710 | 5.282 | 2.820 |
|  | b_1_ | Pearson Correlation | 0.867 | 1 | -0.045 | 0.178 | -0.999 |
|  |  | Bayes Factor | 0.000 |  | 7.464 | 4.260 | 0.029 |
|  | b_2_ | Pearson Correlation | 0.008 | -0.045 | 1 | -0.544 | 0.396 |
|  |  | Bayes Factor | 7.710 | 7.464 |  | 0.576 | 2.463 |
|  | b_3_ | Pearson Correlation | 0.020 | 0.178 | -0.544 | 1 | 1 |
|  |  | Bayes Factor | 5.282 | 4.260 | 0.576 |  | 0.001 |
|  | b_4_ | Pearson Correlation | 0.216 | -0.999 | 0.396 | 1 | 1 |
|  |  | Bayes Factor | 2.820 | 0.029 | 2.463 | 0.001 |  |
