# Supplementary material for: Goat Milk Nutritional Quality Software-Automatized Individual Curve Model Fitting, Shape Parameters Calculation and Bayesian Flexibility Criteria Comparison
Source: Animals (Basel). 2020 Sep 18;10(9):1693. doi: 10.3390/ani10091693 (PMC7552780; doi:10.3390/ani10091693)
Supplement: Supplementary file 1 [file animals-10-01693-s001.zip › Table S7.docx]

**Table S7:** Summary of curve shape parameters (b0, b1, b2, b3, b4 and knot), number of elements and flexibility selection criterion (RSS, AIC, AICc and BIC) for linear and non-linear models for milk protein content in Murciano-Granadina goats.

| **Model name** | **b0** | **b1** | **b2** | **b3** | **b4** | **Knot** | **Elements** | **RSS** | **MSPE** | **AIC** | **AICc** | **BIC** |
| --- | --- | --- | --- | --- | --- | --- | --- | --- | --- | --- | --- | --- |
| Ali and Schaeffer model (ALISCH) | 4.62 | 0.01 | 0.00 | 1.06 | 0.10 | NA | 5 | 728.57 | 145.71 | 34.52 | 39.09 | 34.46 |
| Asymptotic Regression, Single Exponential decay to an arbitrary value (SXPDCY) | 3.43 | 0.00 | NA | NA | NA | NA | 2 | 767.35 | 383.68 | 34.88 | 39.45 | 34.83 |
| Asymptotic Regression, Lactation modification of Metcherlich Law of Diminishing Returns or Exponential growth model (METLAW) | 3.23 | -0.29 | 0.06 | 0.00 | NA | NA | 4 | 729.84 | 182.46 | 34.53 | 39.10 | 34.47 |
| Brody (BRODY) | 3.43 | 0.00 | 15.50 | NA | NA | NA | 3 | 767.35 | 255.78 | 34.88 | 39.45 | 34.83 |
| Cappio Borlino, biexponential (CAPBOR) | 4.44 | -0.08 | 0.00 | NA | NA | NA | 3 | 730.12 | 243.37 | 34.53 | 39.10 | 34.48 |
| Cobby and Le Du (COBLDU) | 4.43 | 0.00 | 0.00 | NA | NA | NA | 3 | 767.59 | 255.86 | 34.88 | 39.45 | 34.83 |
| Compound/ Exponential Growth (CEXPGR) | 5.19 | 1.00 | NA | NA | NA | NA | 2 | 4126.97 | 2063.48 | 46.66 | 51.23 | 46.60 |
| Cubic (CUBIC) | 3.80 | -0.01 | 0.00 | 0.00 | NA | NA | 4 | 738.65 | 184.66 | 34.61 | 39.18 | 34.56 |
| Cubic Spline function with one knot (CUBSPL) | -24.55 | 1.56 | -0.03 | 0.00 | 0.00 | 54.06 | 5 | 738.65 | 147.73 | 34.61 | 39.18 | 34.56 |
| Curve S (CURVES) | 1.26 | 0.39 | NA | NA | NA | NA | 2 | 764.87 | 382.44 | 34.86 | 39.43 | 34.80 |
| Density (DENSITY) | NA | NA | NA | NA | NA | NA | 3 | NA | NA | NA | NA | NA |
| Dhanoa (DHANOA) | 3.80 | 0.00 | 0.01 | NA | NA | NA | 3 | 741.43 | 247.14 | 34.64 | 39.21 | 34.58 |
| Dijkstra (DJKSTR) | -0.08 | 0.08 | 0.01 | -0.09 | NA | NA | 4 | 3456.69 | 864.17 | 45.42 | 49.99 | 45.36 |
| Exponential decline function or Gaines (EDFGAIN) | 3.43 | 0.00 | NA | NA | NA | NA | 2 | 767.35 | 383.68 | 34.88 | 39.45 | 34.83 |
| Gauss (GAUSS) | NC | NC | NC | NA | NA | NA | 3 | NC | NC | NC | NC | NC |
| Gompertz (GMPRTZ) | 90.91 | 3.28 | 0.00 | NA | NA | NA | 3 | 767.43 | 255.81 | 34.88 | 39.45 | 34.83 |
| Grossman (GROSMN) | 4.45 | -0.08 | 0.00 | 0.01 | 0.01 | NA | 5 | 728.91 | 145.78 | 34.52 | 39.09 | 34.47 |
| Hayashi (HAYSHI) | 71938482.20 | 14.48 | -0.04 | NA | NA | NA | 3 | 4144.63 | 1381.54 | 46.69 | 51.26 | 46.63 |
| Inverse quadratic polynomial (INVQPOL) | -6534.83 | 430.69 | -2.71 | NA | NA | NA | 3 | 39263.65 | 13087.88 | 62.43 | 67.00 | 62.37 |
| Inverse, linear Hyperbolic (INVLINHY) | 3.51 | 1.48 | NA | NA | NA | NA | 2 | 765.52 | 382.76 | 34.86 | 39.43 | 34.81 |
| Johnson Schumacher (JOHNSCH) | 3.52 | -0.28 | -0.39 | NA | NA | NA | 3 | 764.07 | 254.69 | 34.85 | 39.42 | 34.80 |
| Log Logistic (LOGLOG) | 3.59 | -1.25 | -0.07 | NA | NA | NA | 3 | 0.00 | 0.00 | NA | NA | NA |
| Log Modified Weibull (LGMWEIB) | -5887.54 | 113.44 | 2.10 | NA | NA | NA | 3 | 0.00 | 0.00 | NA | NA | NA |
| Logarithmic (LOGARITH) | 3.50 | 0.01 | NA | NA | NA | NA | 2 | 779.04 | 389.52 | 34.99 | 39.56 | 34.93 |
| Madalena (MADALN) | 3.43 | 0.00 | NA | NA | NA | NA | 2 | 767.59 | 383.79 | 34.88 | 39.45 | 34.83 |
| Michaelis Menten (MICHMEN) | NA | NC | NC | NA | NA | NA | 2 | NC | NC | NC | NC | NC |
| MilkBot (MILKBOT) | 124.74 | 0.02 | 0.00 | NA | NA | NA | 3 | 39678.87 | 13226.29 | 62.50 | 67.07 | 62.44 |
| Molina and Boschini/Modal Linear (MOL&BOS) | 3.39 | 0.00 | 81.26 | NA | NA | NA | 3 | 746.44 | 248.81 | 34.69 | 39.26 | 34.63 |
| Morgan Mercer Florin (MORMFLO) | 3.90 | 0.00 | 3.47 | -4.91 | NA | NA | 4 | 757.80 | 189.45 | 34.79 | 39.36 | 34.74 |
| Nelder, inverser polynomial, Yadav (NELDER) | -3744.66 | 401.14 | -2.64 | NA | NA | NA | 3 | 39421.86 | 13140.62 | 62.45 | 67.02 | 62.40 |
| Parabolic exponential model and Parabolic, Sikka (PEMSIK) | 3.62 | 0.00 | 0.00 | NA | NA | NA | 3 | 750.21 | 250.07 | 34.72 | 39.29 | 34.67 |
| Parabolic yield-density (PARYLDENS) | -1344.46 | 22.92 | -0.10 | NA | NA | NA | 3 | 39409.87 | 13136.62 | 62.45 | 67.02 | 62.40 |
| Power (POWER) | 3.50 | 0.00 | NA | NA | NA | NA | 2 | 779.02 | 389.51 | 34.98 | 39.56 | 34.93 |
| Quadratic cum log model (QDCMLOG) | 4.54 | 0.01 | 0.00 | -0.38 | NA | NA | 4 | 728.62 | 182.16 | 34.52 | 39.09 | 34.46 |
| Quadratic model (QUADRT) | 3.62 | 0.00 | 0.00 | NA | NA | NA | 3 | 749.88 | 249.96 | 34.72 | 39.29 | 34.66 |
| Quadratic model Dave (DAVE) | 3.62 | 0.00 | 0.00 | NA | NA | NA | 3 | 749.88 | 249.96 | 34.72 | 39.29 | 34.66 |
| Quadratic spline function with one knot (QUADSPL) | 3.52 | 0.00 | 0.00 | 0.00 | NA | 51.15 | 4 | 749.88 | 187.47 | 34.72 | 39.29 | 34.66 |
| Ratio Cubics/Partial Fraction with Cubic Denominator (RATCUB) | 0.00 | 0.00 | 0.00 | 0.00 | 0.00 | NA | 5 | 762.59 | 152.52 | 34.84 | 39.41 | 34.78 |
| Ratio Quadratics/Partial Fraction with Quadratic Denominator (RATQUAD) | 0.14 | 0.06 | 0.32 | 0.09 | NA | NA | 4 | 763.67 | 190.92 | 34.85 | 39.42 | 34.79 |
| Richards (RICHRDS) | 3.52 | 0.15 | -1.35 | 7.14 | NA | NA | 4 | 1143.25 | 285.81 | 37.67 | 42.24 | 37.62 |
| Rook (ROOK) | 0.00 | -3.91 | 0.00 | 0.00 | NA | NA | 4 | 767.35 | 191.84 | 34.88 | 39.45 | 34.83 |
| Simple Linear (SIMLIN) | 3.43 | 0.00 | NA | NA | NA | NA | 2 | 767.59 | 383.79 | 34.88 | 39.45 | 34.83 |
| Singh And Gopal (SIN&GOP) | 4.37 | 0.00 | -0.30 | NA | NA | NA | 3 | 730.27 | 243.42 | 34.53 | 39.10 | 34.48 |
| Third order Legendre ortogonal polynomial (3ORDLEG) | -125.68 | 269.38 | -115.25 | 119.26 | NA | NA | 4 | 16546.94 | 4136.73 | 56.38 | 60.95 | 56.32 |
| Verhulst/Logistic differential equation/Pearl Reed (VERHLST) | 51045.64 | 14880.03 | 0.00 | NA | NA | NA | 3 | 767.35 | 255.78 | 34.88 | 39.45 | 34.83 |
| Von Bertalanffy (VBRTLNFY) | 3.54 | 0.92 | 0.04 | NA | NA | NA | 3 | 779.26 | 259.75 | 34.99 | 39.56 | 34.93 |
| Weibull, Parametric Survival Models (PARSURW) | 3.11 | -0.43 | -1.75 | -2038.08 | NA | NA | 4 | 770.99 | 192.75 | 34.91 | 39.48 | 34.86 |
| Wilmink’s exponential (WILMINK) | 3.21 | 0.91 | 0.00 | NA | NA | NA | 3 | 730.06 | 243.35 | 34.53 | 39.10 | 34.48 |
| Wood (WOOD) | 3.21 | -0.91 | NA | 0.00 | NA | NA | 3 | 730.06 | 243.35 | 34.53 | 39.10 | 34.48 |
| NC: Does not converge; NA: Does not apply. | | | | | | | | | | | | |
