# Supplementary material for: Goat Milk Nutritional Quality Software-Automatized Individual Curve Model Fitting, Shape Parameters Calculation and Bayesian Flexibility Criteria Comparison
Source: Animals (Basel). 2020 Sep 18;10(9):1693. doi: 10.3390/ani10091693 (PMC7552780; doi:10.3390/ani10091693)
Supplement: Supplementary file 1 [file animals-10-01693-s001.zip › Table S8.docx]

**Table S8:** Summary of curve shape parameters (b0, b1, b2, b3, b4 and knot), number of elements and flexibility selection criterion (RSS, AIC, AICc and BIC) for linear and non-linear models for milk fat content in Murciano-Granadina goats.

| **Model name** | **b0** | **b1** | **b2** | **b3** | **b4** | **Knot** | **Elements** | **RSS** | **MSPE** | **AIC** | **AICc** | **BIC** |
| --- | --- | --- | --- | --- | --- | --- | --- | --- | --- | --- | --- | --- |
| Ali and Schaeffer model (ALISCH) | 4.81 | 0.01 | 0.00 | -1.58 | -0.97 | NA | 5 | 4078.42 | 815.68 | 51.14 | 46.52 | 46.57 |
| Asymptotic Regression, Single Exponential decay to an arbitrary value (SXPDCY) | 5.19 | 0.00 | NA | NA | NA | NA | 2 | 4126.97 | 2063.49 | 51.23 | 46.60 | 46.66 |
| Asymptotic Regression, Lactation modification of Metcherlich Law of Diminishing Returns or Exponential growth model (METLAW) | 4.05 | -0.38 | 0.01 | 0.00 | NA | NA | 4 | 4080.04 | 1020.01 | 51.15 | 46.52 | 46.58 |
| Brody (BRODY) | 5.19 | 0.00 | 15.94 | NA | NA | NA | 3 | 4126.97 | 1375.66 | 51.23 | 46.60 | 46.66 |
| Cappio Borlino, biexponential (CAPBOR) | 6.07 | -0.05 | 0.00 | NA | NA | NA | 3 | 4095.64 | 1365.21 | 51.17 | 46.55 | 46.60 |
| Cobby and Le Du (COBLDU) | 6.20 | 0.00 | 0.00 | NA | NA | NA | 3 | 4127.33 | 1375.78 | 51.23 | 46.60 | 46.66 |
| Compound/ Exponential Growth (CEXPGR) | 2.24 | 1.00 | NA | NA | NA | NA | 2 | 56202.33 | 28101.17 | 69.51 | 64.88 | 64.94 |
| Cubic (CUBIC) | 5.58 | -0.01 | 0.00 | 0.00 | NA | NA | 4 | 4079.85 | 1019.96 | 51.15 | 46.52 | 46.58 |
| Cubic Spline function with one knot (CUBSPL) | -828.24 | 14.77 | -0.09 | 0.00 | 0.00 | 169.29 | 5 | 4079.85 | 815.97 | 51.15 | 46.52 | 46.58 |
| Curve S (CURVES) | 1.67 | 0.04 | NA | NA | NA | NA | 2 | 4144.63 | 2072.32 | 51.26 | 46.63 | 46.69 |
| Density (DENSITY) | NC | NC | NC | NA | NA | NA | 3 | NC | NC | NC | NC | NC |
| Dhanoa (DHANOA) | 5.68 | 0.00 | 0.01 | NA | NA | NA | 3 | 4081.82 | 1360.61 | 51.15 | 46.52 | 46.58 |
| Dijkstra (DJKSTR) | -0.13 | 0.08 | 0.01 | -0.14 | NA | NA | 4 | 9991.93 | 2497.98 | 57.42 | 52.79 | 52.85 |
| Exponential decline function or Gaines (EDFGAIN) | 5.19 | 0.00 | NA | NA | NA | NA | 2 | 4126.97 | 2063.49 | 51.23 | 46.60 | 46.66 |
| Gauss (GAUSS) | 5.20 | 0.00 | -0.01 | NA | NA | NA | 3 | 4103.62 | 1367.87 | 51.19 | 46.56 | 46.62 |
| Gompertz (GMPRTZ) | 5.32 | -0.04 | 0.06 | NA | NA | NA | 3 | 4141.94 | 1380.65 | 51.25 | 46.63 | 46.68 |
| Grossman (GROSMN) | 6.08 | -0.05 | 0.00 | 0.01 | 0.01 | NA | 5 | 4091.47 | 818.29 | 51.17 | 46.54 | 46.60 |
| Hayashi (HAYSHI) | 71938482.20 | 14.48 | -0.04 | NA | NA | NA | 3 | 4144.63 | 1381.54 | 51.26 | 46.63 | 46.69 |
| Inverse quadratic polynomial (INVQPOL) | -10897.45 | 330.36 | -1.79 | NA | NA | NA | 3 | 91491.12 | 30497.04 | 72.92 | 68.29 | 68.35 |
| Inverse, linear Hyperbolic (INVLINHY) | 5.33 | 0.19 | NA | NA | NA | NA | 2 | 4144.63 | 2072.32 | 51.26 | 46.63 | 46.69 |
| Johnson Schumacher (JOHNSCH) | 5.33 | 0.01 | -15.11 | NA | NA | NA | 3 | 4138.33 | 1379.44 | 51.25 | 46.62 | 46.67 |
| Log Logistic (LOGLOG) | 5.30 | -4.64 | 0.17 | NA | NA | NA | 3 | 4063.45 | 1354.48 | 51.12 | 46.49 | 46.55 |
| Log Modified Weibull (LGMWEIB) | NC | NC | NC | NA | NA | NA | 3 | NC | NC | NC | NC | NC |
| Logarithmic (LOGARITH) | 5.20 | 0.03 | NA | NA | NA | NA | 2 | 4143.02 | 2071.51 | 51.25 | 46.63 | 46.68 |
| Madalena (MADALN) | 5.20 | 0.00 | NA | NA | NA | NA | 2 | 4127.33 | 2063.67 | 51.23 | 46.60 | 46.66 |
| Michaelis Menten (MICHMEN) | NA | NC | NC | NA | NA | NA | 2 | NC | NC | NC | NC | NC |
| MilkBot (MILKBOT) | 121.20 | 0.02 | 0.00 | NA | NA | NA | 3 | 92342.20 | 30780.73 | 72.98 | 68.36 | 68.41 |
| Molina and Boschini/Modal Linear (MOL&BOS) | 5.09 | 0.00 | 97.35 | NA | NA | NA | 3 | 4077.77 | 1359.26 | 51.14 | 46.52 | 46.57 |
| Morgan Mercer Florin (MORMFLO) | 6.80 | 0.00 | 5.25 | -5.14 | NA | NA | 4 | 4101.44 | 1025.36 | 51.18 | 46.56 | 46.61 |
| Nelder, inverser polynomial, Yadav (NELDER) | -16270.88 | 446.81 | -2.34 | NA | NA | NA | 3 | 91460.16 | 30486.72 | 72.92 | 68.29 | 68.34 |
| Parabolic exponential model and Parabolic, Sikka (PEMSIK) | 5.48 | 0.00 | 0.00 | NA | NA | NA | 3 | 4083.64 | 1361.21 | 51.15 | 46.53 | 46.58 |
| Parabolic yield-density (PARYLDENS) | -223.21 | 4.77 | -0.02 | NA | NA | NA | 3 | 91695.39 | 30565.13 | 72.93 | 68.31 | 68.36 |
| Power (POWER) | 5.20 | 0.01 | - | NA | NA | NA | 2 | 4142.97 | 2071.49 | 51.25 | 46.63 | 46.68 |
| Quadratic cum log model (QDCMLOG) | 5.58 | -0.01 | 0.00 | -0.04 | NA | NA | 4 | 4083.01 | 1020.75 | 51.15 | 46.53 | 46.58 |
| Quadratic model (QUADRT) | 5.49 | -0.01 | 0.00 | NA | NA | NA | 3 | 4083.24 | 1361.08 | 51.15 | 46.53 | 46.58 |
| Quadratic model Dave (DAVE) | 5.49 | -0.01 | 0.00 | NA | NA | NA | 3 | 4083.24 | 1361.08 | 51.15 | 46.53 | 46.58 |
| Quadratic spline function with one knot (QUADSPL) | 4.98 | 0.01 | 0.00 | 0.00 | NA | 52.71 | 4 | 4083.24 | 1020.81 | 51.15 | 46.53 | 46.58 |
| Ratio Cubics/Partial Fraction with Cubic Denominator (RATCUB) | 0.00 | -0.05 | 0.04 | 0.36 | 0.07 | NA | 5 | 4144.28 | 828.86 | 51.26 | 46.63 | 46.69 |
| Ratio Quadratics/Partial Fraction with Quadratic Denominator (RATQUAD) | 0.00 | 0.00 | 0.01 | 0.00 | NA | NA | 4 | 4144.28 | 1036.07 | 51.26 | 46.63 | 46.69 |
| Richards (RICHRDS) | 5.32 | 0.07 | 0.32 | -5.93 | NA | NA | 4 | 4141.89 | 1035.47 | 51.25 | 46.63 | 46.68 |
| Rook (ROOK) | 0.00 | -3.89 | 0.00 | 0.00 | NA | NA | 4 | 4126.97 | 1031.74 | 51.23 | 46.60 | 46.66 |
| Simple Linear (SIMLIN) | 5.20 | 0.00 | NA | NA | NA | NA | 2 | 4127.33 | 2063.67 | 51.23 | 46.60 | 46.66 |
| Singh And Gopal (SIN&GOP) | 6.06 | 0.00 | -0.28 | NA | NA | NA | 3 | 4095.17 | 1365.06 | 51.17 | 46.55 | 46.60 |
| Third order Legendre ortogonal polynomial (3ORDLEG) | -345.00 | -279.24 | -313.67 | -120.18 | NA | NA | 4 | 19177.00 | 4794.25 | 61.98 | 57.35 | 57.41 |
| Verhulst/Logistic differential equation/Pearl Reed (VERHLST) | 5.32 | -0.04 | 0.06 | NA | NA | NA | 3 | 4141.96 | 1380.65 | 51.25 | 46.63 | 46.68 |
| Von Bertalanffy (VBRTLNFY) | 5.32 | -0.01 | 0.06 | NA | NA | NA | 3 | 4141.93 | 1380.64 | 51.25 | 46.63 | 46.68 |
| Weibull, Parametric Survival Models (PARSURW) | 5.21 | -0.12 | 197.59 | -2408.54 | NA | NA | 4 | 4144.82 | 1036.21 | 51.26 | 46.63 | 46.69 |
| Wilmink’s exponential (WILMINK) | 4.99 | 0.83 | 0.00 | NA | NA | NA | 3 | 4096.23 | 1365.41 | 51.17 | 46.55 | 46.60 |
| Wood (WOOD) | 4.99 | -0.83 | NA | 0.00 | NA | NA | 3 | 4096.23 | 1365.41 | 51.17 | 46.55 | 46.60 |
| NC: Does not converge; NA: Does not apply, | | | | | | | | | | | | |
