# Supplementary material for: Goat Milk Nutritional Quality Software-Automatized Individual Curve Model Fitting, Shape Parameters Calculation and Bayesian Flexibility Criteria Comparison
Source: Animals (Basel). 2020 Sep 18;10(9):1693. doi: 10.3390/ani10091693 (PMC7552780; doi:10.3390/ani10091693)
Supplement: Supplementary file 1 [file animals-10-01693-s001.zip › Table S9.docx]

**Table S9:** Summary of curve shape parameters (b0, b1, b2, b3, b4 and knot), number of elements and flexibility selection criterion (RSS, AIC, AICc and BIC) for linear and non-linear models for milk dry matter content in Murciano-Granadina goats.

| **Model name** | **b0** | **b1** | **b2** | **b3** | **b4** | **Knot** | **Elements** | **RSS** | **MSPE** | **AIC** | **AICc** | **BIC** |
| --- | --- | --- | --- | --- | --- | --- | --- | --- | --- | --- | --- | --- |
| Ali and Schaeffer model (ALISCH) | 14.97 | 0.01 | 0.00 | -0.92 | -0.99 | NA | 5 | 6279.22 | 1255.84 | 49.59 | 54.17 | 49.54 |
| Asymptotic Regression, Single Exponential decay to an arbitrary value (SXPDCY) | 14.38 | 0.00 | NA | NA | NA | NA | 2 | 6457.30 | 3228.65 | 49.79 | 54.36 | 49.74 |
| Asymptotic Regression, Lactation modification of Metcherlich Law of Diminishing Returns or Exponential growth model (METLAW) | 13.17 | -0.16 | 0.02 | 0.00 | NA | NA | 4 | 6274.70 | 1568.68 | 49.59 | 54.16 | 49.53 |
| Brody (BRODY) | 14.38 | 0.00 | 18.65 | NA | NA | NA | 3 | 6457.30 | 2152.43 | 49.79 | 54.36 | 49.74 |
| Cappio Borlino, biexponential (CAPBOR) | 16.36 | -0.04 | 0.00 | NA | NA | NA | 3 | 6301.36 | 2100.45 | 49.62 | 54.19 | 49.56 |
| Cobby and Le Du (COBLDU) | 15.38 | 0.00 | 0.00 | NA | NA | NA | 3 | 6457.37 | 2152.46 | 49.79 | 54.36 | 49.74 |
| Compound/ Exponential Growth (CEXPGR) | 3.43 | 1.00 | NA | NA | NA | NA | 2 | 767.35 | 383.68 | 34.88 | 39.45 | 34.83 |
| Cubic (CUBIC) | 15.19 | -0.02 | 0.00 | 0.00 | NA | NA | 4 | 6277.25 | 1569.31 | 49.59 | 54.16 | 49.54 |
| Cubic Spline function with one knot (CUBSPL) | 23.70 | 0.62 | 0.02 | 0.00 | 0.00 | -39.60 | 5 | 6277.25 | 1255.45 | 49.59 | 54.16 | 49.54 |
| Curve S (CURVES) | 2.67 | 0.17 | NA | NA | NA | NA | 2 | 6421.82 | 3210.91 | 49.75 | 54.32 | 49.70 |
| Density (DENSITY) | NC | NC | NC | NA | NA | NA | 3 | NC | NC | NC | NC | NC |
| Dhanoa (DHANOA) | 15.34 | 0.00 | 0.00 | NA | NA | NA | 3 | 6280.18 | 2093.39 | 49.59 | 54.17 | 49.54 |
| Dijkstra (DJKSTR) | -0.35 | 0.08 | 0.01 | -0.38 | NA | NA | 4 | 50248.78 | 12562.20 | 64.15 | 68.72 | 64.10 |
| Exponential decline function or Gaines (EDFGAIN) | 14.38 | 0.00 | NA | NA | NA | NA | 2 | 6457.30 | 3228.65 | 49.79 | 54.36 | 49.74 |
| Gauss (GAUSS) | 14.35 | 0.00 | 0.00 | NA | NA | NA | 3 | 6410.45 | 2136.82 | 49.74 | 54.31 | 49.68 |
| Gompertz (GMPRTZ) | 14.40 | -0.07 | 0.06 | NA | NA | NA | 3 | 6395.72 | 2131.91 | 49.72 | 54.29 | 49.67 |
| Grossman (GROSMN) | 16.38 | -0.04 | 0.00 | 0.00 | 0.01 | NA | 5 | 6291.29 | 1258.26 | 49.61 | 54.18 | 49.55 |
| Hayashi (HAYSHI) | 541504420.36 | 39.17 | -0.17 | NA | NA | NA | 3 | 6421.82 | 2140.61 | 49.75 | 54.32 | 49.70 |
| Inverse quadratic polynomial (INVQPOL) | -905.02 | 96.95 | -0.64 | NA | NA | NA | 3 | 650309.75 | 216769.92 | 82.08 | 86.65 | 82.02 |
| Inverse, linear Hyperbolic (INVLINHY) | 14.41 | 2.56 | NA | NA | NA | NA | 2 | 6421.13 | 3210.57 | 49.75 | 54.32 | 49.70 |
| Johnson Schumacher (JOHNSCH) | 14.26 | 2.02 | -300.53 | NA | NA | NA | 3 | 6423.01 | 2141.00 | 49.75 | 54.32 | 49.70 |
| Log Logistic (LOGLOG) | 14.39 | -20.13 | 0.14 | NA | NA | NA | 3 | 9762.13 | 3254.04 | 52.68 | 57.25 | 52.63 |
| Log Modified Weibull (LGMWEIB) | -5457.25 | 122.15 | 2.11 | NA | NA | NA | 3 | 0.00 | 0.00 | NA | NA | NA |
| Logarithmic (LOGARITH) | 14.77 | -0.07 | NA | NA | NA | NA | 2 | 6451.44 | 3225.72 | 49.78 | 54.35 | 49.73 |
| Madalena (MADALN) | 14.38 | 0.00 | NA | NA | NA | NA | 2 | 6457.37 | 3228.69 | 49.79 | 54.36 | 49.74 |
| Michaelis Menten (MICHMEN) | NA | NA | NA | NA | NA | NA | 2 | NC | NC | NC | NC | NC |
| MilkBot (MILKBOT) | 376.89 | 0.02 | 0.00 | NA | NA | NA | 3 | 655099.34 | 218366.45 | 82.13 | 86.70 | 82.07 |
| Molina and Boschini/Modal Linear (MOL&BOS) | 14.08 | -0.01 | 109.55 | NA | NA | NA | 3 | 6306.32 | 2102.11 | 49.62 | 54.20 | 49.57 |
| Morgan Mercer Florin (MORMFLO) | 15.25 | 223.76 | 14.40 | 2.05 | NA | NA | 4 | 6402.81 | 1600.70 | 49.73 | 54.30 | 49.68 |
| Nelder, inverser polynomial, Yadav (NELDER) | -905.03 | 96.95 | -0.64 | NA | NA | NA | 3 | 650309.75 | 216769.92 | 82.08 | 86.65 | 82.02 |
| Parabolic exponential model and Parabolic, Sikka (PEMSIK) | 14.94 | 0.00 | 0.00 | NA | NA | NA | 3 | 6301.37 | 2100.46 | 49.62 | 54.19 | 49.56 |
| Parabolic yield-density (PARYLDENS) | -682.26 | 10.40 | -0.04 | NA | NA | NA | 3 | 650231.49 | 216743.83 | 82.07 | 86.65 | 82.02 |
| Power (POWER) | 14.78 | -0.01 | NA | NA | NA | NA | 2 | 6451.23 | 3225.62 | 49.78 | 54.35 | 49.73 |
| Quadratic cum log model (QDCMLOG) | 15.76 | 0.00 | 0.00 | -0.34 | NA | NA | 4 | 6284.00 | 1571.00 | 49.60 | 54.17 | 49.54 |
| Quadratic model (QUADRT) | 14.93 | -0.01 | 0.00 | NA | NA | NA | 3 | 6301.11 | 2100.37 | 49.62 | 54.19 | 49.56 |
| Quadratic model Dave (DAVE) | 14.93 | -0.01 | 0.00 | NA | NA | NA | 3 | 6301.11 | 2100.37 | 49.62 | 54.19 | 49.56 |
| Quadratic spline function with one knot (QUADSPL) | 12.14 | 0.05 | 0.00 | 0.00 | NA | 97.54 | 4 | 6301.11 | 1575.28 | 49.62 | 54.19 | 49.56 |
| Ratio Cubics/Partial Fraction with Cubic Denominator (RATCUB) | 5.45 | -7.61 | 3.50 | 9.16 | 0.64 | NA | 5 | 6414.79 | 1282.96 | 49.74 | 54.31 | 49.69 |
| Ratio Quadratics/Partial Fraction with Quadratic Denominator (RATQUAD) | 0.00 | 0.00 | 0.00 | 0.00 | NA | NA | 4 | 6418.24 | 1604.56 | 49.75 | 54.32 | 49.69 |
| Richards (RICHRDS) | 14.40 | 0.06 | 0.00 | -0.05 | NA | NA | 4 | 6395.72 | 1598.93 | 49.72 | 54.29 | 49.67 |
| Rook (ROOK) | 0.00 | -3.80 | 0.00 | 0.00 | NA | NA | 4 | 6457.30 | 1614.33 | 49.79 | 54.36 | 49.74 |
| Simple Linear (SIMLIN) | 14.38 | 0.00 | NA | NA | NA | NA | 2 | 6457.37 | 3228.69 | 49.79 | 54.36 | 49.74 |
| Singh And Gopal (SIN&GOP) | 16.31 | -0.01 | 0.61 | NA | NA | NA | 3 | 6299.57 | 2099.86 | 49.62 | 54.19 | 49.56 |
| Third order Legendre ortogonal polynomial (3ORDLEG) | -126.90 | -312.02 | -130.16 | -134.46 | NA | NA | 4 | 20629.40 | 5157.35 | 57.92 | 62.49 | 57.87 |
| Verhulst/Logistic differential equation/Pearl Reed (VERHLST) | 14.40 | -0.07 | 0.06 | NA | NA | NA | 3 | 6395.89 | 2131.96 | 49.72 | 54.29 | 49.67 |
| Von Bertalanffy (VBRTLNFY) | 14.40 | -0.02 | 0.06 | NA | NA | NA | 3 | 6395.67 | 2131.89 | 49.72 | 54.29 | 49.67 |
| Weibull, Parametric Survival Models (PARSURW) | 16.44 | 1.99 | 51.55 | -19652.13 | NA | NA | 4 | 6454.64 | 1613.66 | 49.79 | 54.36 | 49.73 |
| Wilmink’s exponential (WILMINK) | 13.93 | 1.83 | 0.00 | NA | NA | NA | 3 | 6305.47 | 2101.82 | 49.62 | 54.19 | 49.57 |
| Wood (WOOD) | 13.93 | -1.83 | NA | 0.00 | NA | NA | 3 | 6305.47 | 2101.82 | 49.62 | 54.19 | 49.57 |
| NC: Does not converge; NA: Does not apply. | | | | | | | | | | | | |
